# Reproducible microbiome composition signatures of anxiety and depressive symptoms

**DOI:** 10.1016/j.csbj.2023.10.035

**Published:** 2023-10-18

**Authors:** Afroditi Kouraki, Anthony Kelly, Amrita Vijay, Sameer Gohir, Stuart Astbury, Vasileios Georgopoulos, Bonnie Millar, David Andrew Walsh, Eamonn Ferguson, Cristina Menni, Ana M. Valdes

**Affiliations:** a Academic Unit of Injury, Recovery and Inflammation Sciences, Rheumatology, School of Medicine, University of Nottingham, Nottingham, UK; b NIHR Nottingham Biomedical Research Centre, Nottingham University Hospitals NHS Trust and the University of Nottingham, Nottingham, UK; c Department of Twin Research and Genetic Epidemiology, King’s College London, London, UK; d Nottingham Digestive Diseases Centre, Translational Medical Sciences, School of Medicine, University of Nottingham, Nottingham, UK; e Pain Centre Versus Arthritis, University of Nottingham, Nottingham, UK; f School of Psychology, University of Nottingham, University Park, Nottingham, UK; g National Institute for Health and Care Research Blood and Transplant Research Unit in Donor Health and Behaviour, Department of Public Health and Primary Care, University of Cambridge, Cambridge, UK

**Keywords:** Anxiety symptoms, Depressive symptoms, Gut microbiota, Fibre, Chronic pain, Meta-analysis

## Abstract

The gut microbiome is a significant contributor to mental health, with growing evidence linking its composition to anxiety and depressive disorders. Gut microbiome composition is associated with signs of anxiety and depression both in clinically diagnosed mood disorders and subclinically in the general population and may be influenced by dietary fibre intake and the presence of chronic pain. We provide an update of current evidence on the role of gut microbiome composition in depressive and anxiety disorders or symptoms by reviewing available studies. Analysing data from three independent cohorts (osteoarthritis 1 (OA1); n = 46, osteoarthritis 2 (OA2); n = 58, and healthy controls (CON); n = 67), we identified microbial composition signatures of anxiety and depressive symptoms at genus level and cross-validated our findings performing meta-analyses of our results with results from previously published studies. The genera *Bifidobacterium* (fixed-effect beta (95% CI) = −0.22 (−0.34, −0.10), p = 3.90e-04) and *Lachnospiraceae NK4A136 group* (fixed-effect beta (95% CI) = −0.09 (−0.13, −0.05), p = 2.53e-06) were found to be the best predictors of anxiety and depressive symptoms, respectively, across our three cohorts and published literature taking into account demographic and lifestyle covariates, such as fibre intake. The association with anxiety was robust in accounting for heterogeneity between cohorts and supports previous observations of the potential prophylactic effect of *Bifidobacterium* against anxiety symptoms.

## Highlights

• Symptoms of anxiety and depression are associated with the gut microbiota.
• Five bacterial genera are associated with anxiety and depressive symptoms.
• Associations were stronger in two chronic pain cohorts than in controls.
• A meta-analysis found *Bifidobacterium* associated with lower anxiety symptoms.
• Our results agree with observations of probiotic supplementation reducing anxiety.

## Graphical Abstract

**Figure:**
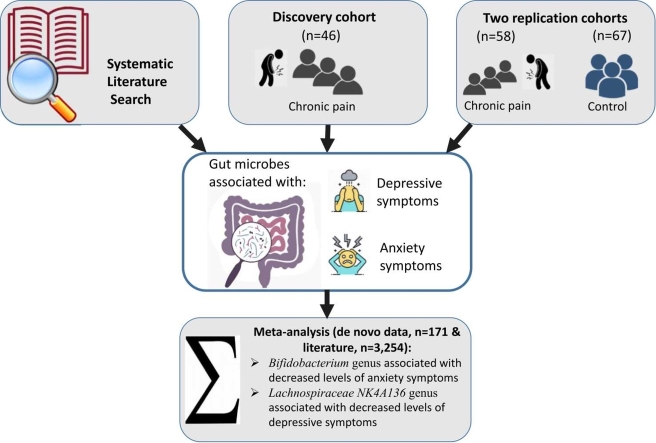

## 1. Introduction

Depression and anxiety are mood disorders that affect 4.7% and 7.3% of the global population, respectively [1], [2] and are a leading cause of disability and mortality [3]. An increasing body of research in recent years has revealed a relationship between the composition of the gut microbiome and mood disorders [4], [5], [6], [7] which has the potential of leading to novel treatment avenues and strategies for effectively managing and treating these conditions [8].

The impact of anxiety and depression on the gut-brain axis has been demonstrated in preclinical models [9] whereby administration of probiotics, such as Bifidobacterium strains, reversed changes in the gut microbiome along with changes in anxiety- and depressive-like behaviour (see [10] for review). Although research in humans had been relatively slow until recently, several studies have highlighted alterations in gut microbiota composition in individuals with clinical depressive disorders compared to healthy controls [11], [12], [13], [14], [15].

A number of systematic reviews have explored the links between the gut microbiota and mood related clinical conditions [16], [17], [18], such as major depressive disorder (MDD) and generalised anxiety disorder (GAD) which represent specific categories of mental illnesses [19]. These reviews include a meta-analysis in MDD performed on alpha diversity indices [15]. However, mental health exists on a spectrum, and many individuals may experience subthreshold symptoms or have undiagnosed conditions [19].

Although several studies have reported an association between anxiety and gut microbiome composition [20], [21], [22], [23], [24], [25], [26], [27], consistent evidence of microbial composition signatures of anxiety and depressive symptoms is still lacking. Previous studies have mostly focused on differential abundance testing [28], and several have not considered the effects of confounders, particularly diet [29]. Identifying consistent microbial features linked to anxiety and depressive symptoms in the general population might enable us to identify critical targets for interventions to alleviate those symptoms.

Both anxiety and depression are more prevalent among individuals who experience chronic pain conditions than among the general population [30], [31]. The presence of chronic pain physical conditions (joint/articular, limb, or back pain, headaches, or gastrointestinal diseases) increases the duration of depressive mood and is much higher among individuals with MDD (40%) than in the general population (17%) [32]. Several studies have indicated gut microbiota alterations among individuals with chronic pain, suggesting a possible role of gut microbiota in chronic pain [33], [34], [35], [36]. In addition, central sensitization which is a major contributor to chronic musculoskeletal pain [37] is also a contributor to inflammatory bowel syndrome and inflammatory bowel disease symptoms [38]. The role of gut microbiota in comorbid chronic pain and depression has only been investigated in a rodent model of neuropathic-like pain, which showed abnormal composition of gut microbiota contributing to individual differences of a depression-like phenotype [39].

There is therefore a need to fill the gap of evidence for the links between gut microbes and anxiety and depressive symptoms in the general population which can provide a more comprehensive understanding of the complex relationship between the gut and the brain. In addition there is a need to understand whether the microbial features associated with anxiety or depressive symptoms in the general population have the same role in individuals with and without chronic pain. Establishing such links may aid in preventing the onset of full-fledged disease and lead to more personalised interventions for individuals with frequently coexisting health issues, such as chronic pain.

The specific aims of the present study are, (i) to systematically search available evidence on gut microbiota composition in depressive and anxiety disorders and symptoms, providing a necessary update of the current literature; (ii) to identify microbial composition signatures of anxiety and depressive symptoms across three independent cohorts of individuals without mood disorders, two of osteoarthritis patients with chronic pain and a control cohort of similar age; (iii) to extend previous published studies by incorporating meta-analyses between anxiety/depressive symptom measures and the gut microbiota; and (iv) to cross-validate our findings combining our new data along with previously published reports, using a prediction-based approach, and taking into account dietary fibre intake and the presence of chronic pain.

## 2. Methods

The study design is outlined in Fig. S1.

### 2.1. Systematic literature search

A systematic search of PubMed was conducted in April 2023 using the search terms: "Gastrointestinal Microbiome"[Mesh] OR Gastrointestinal[tiab] OR gut[tiab] OR intestinal[tiab] AND Microbiota[tiab] OR Microbiome[tiab] OR flora[tiab] AND "Anxiety"[Mesh] OR "Anxiety Disorders"[Mesh] OR "Phobia, Social"[Mesh] OR "Patient Health Questionnaire"[Mesh] OR Anxiety[tiab] OR stress[tiab] OR panic disorder[tiab] OR social anxiety disorder[tiab] OR generali* anxiety disorder[tiab] OR "Gastrointestinal Microbiome"[Mesh] OR Gastrointestinal[tiab] OR gut[tiab] OR intestinal[tiab] AND Microbiota[tiab] OR Microbiome[tiab] OR flora[tiab] AND "Depression"[Mesh] OR "Depressive Disorder"[Mesh] OR "Dysthymic Disorder"[Mesh] OR "Depressive Disorder, Major"[Mesh] OR Depression[tiab] OR Depressive disorder[tiab] OR Major Depression[tiab] OR Major Depressive Disorder[tiab] OR Dysthymic Disorder[tiab] OR Major Dysthymic Disorder[tiab] OR persistent depressive disorder[tiab]. The search yielded 1968 records. Studies were included if they: 1) assessed the gut microbiota composition in anxiety or depressive disorders, or 2) investigated associations between the gut microbiota and anxiety/depression symptom measures in healthy participants or relevant conditions (i.e., anxiety, depressive disorders). They were excluded if they: 1) examined the gut microbiota and anxiety/depression symptoms solely in another psychiatric disorder or disease, 2) assessed the effect of an intervention without reporting relevant baseline measurements. Studies were further excluded if they were: 3) in languages other than English, 4) letters, reports, or abstracts from congresses or symposia, 5) review, protocol and method studies, 6) animals and non-adult studies. The 73 included studies along with relevant findings are summarised in Table S1.

### 2.2. Study populations

Data were taken from baseline visits of three independent study cohorts, iBEAT-OA (OA1), Molecular Pathways (OA2) and Omega-3/Fibre (CON). OA1 and OA2 are knee osteoarthritis cohorts of community dwelling individuals (Age: >45 y) recruited from the Nottinghamshire area [40]. CON is a healthy cohort of participants aged > 18 y that were enrolled from the TwinsUK registry [41]. We only included data from baseline visits in our analysis for the subset of participants for whom gut microbiome composition was available. Participants were included in the final analysis if they had no missing data on anxiety/depressive symptom scores (OA1, n = 46; OA2, n = 67; CON, n = 58). Data were combined with previously published results from Taylor et al. [42] (n = 43) and Bosch et al. [43] (n = 3211) in meta-analysis.

All participants provided written informed consent. For OA1 and OA2, ethical approval was obtained from the East Midlands – Derby NHS Research Ethics Committee (refs: 20/EM/0065, 18/EM/0154, respectively) and the Health Research Authority (protocol no: 19098, 18021). The trials are registered under the clinicaltrials.gov database (NCT04443452, NCT03545048, respectively). For CON the trial was approved by the West Midlands Black Country NHS Research Ethics Committee (18/WM/0066) and is registered under the clinicaltrials.gov database (NCT03442348).

### 2.3. Anxiety and depressive symptom measures

In OA1 and CON, the Hospital Anxiety and Depression Scale Anxiety (HADS-A) and the HADS Depression (HADS-D) subscales were used to measure severity of anxiety and depression symptoms, respectively [44]. Participants were scored based on these subscales from 0 to 21 with higher scores indicating higher anxiety or depression. In OA2, anxiety and depressive symptoms were measured using two validated items originating from the HADS-A and HADS-D, respectively [45], [46]. The items were scored from 1 to 4 with a higher score indicating higher anxiety and depressive symptoms. The studies by Taylor et al. [42] and Bosch et al. [43] included in the meta-analysis (see Results below) utilised the 42-item Depression, Anxiety, and Stress Scale and the 9-item Patient Health Questionnaire-9 to assess the severity of anxiety and depressive symptoms, with higher scores indicating higher anxiety and depressive symptom levels, respectively.

### 2.4. Dietary information

Dietary intake was measured in the OA1 cohort using the validated European Prospective Investigation into Cancer (EPIC) Norfolk Food Frequency Questionnaire (FFQ) version 6 (CAMB/PQ/6/1205) which contains a list of 130 commonly and less commonly consumed foods. Participants are asked to indicate their usual frequency of consumption ranging from “never or less than once/month” to “6 + times per day”. The servings are specified in terms of units or common portions (e.g. one apple, one slice of bread) or household measures (e.g. glass, cup, spoon). Data from the EPIC-Norfolk FFQs were analysed using the FETA software which can be downloaded and used for free upon registration [47]. For each of the 130 items, FETA obtains the nutrient composition of the actual amount eaten by multiplying the nutrient composition per gram by the weighted average daily food intake. The sum of all the FFQ items gives us the average daily nutrient intake for a participant. The recommended fibre intake that was used as covariate in linear regressions with anxiety and depressive symptoms was calculated based on the current dietary reference intake recommendations (14 g ⋅ 1000 kcal−1 ⋅ day−1) [48], [49].

### 2.5. Stool sample collection

Faecal sample collection methods were the same in all cohort studies. Participants were given a stool collection kit and leaflet with detailed instructions on how to collect and post the sample to the assessment centre in the prepaid postbox provided at the end of their baseline visit. All samples were either immediately frozen at − 80 °C or at − 20 °C temporarily until they could be transported to − 80 °C freezers in the Clinical Science Building at City Hospital or at the Queen's Medical Centre following local standard operating procedures (SOPs) prior to analysis. SOPs were used at the collection sites and when transferring the samples which were handled by trained research personnel to ensure the high quality and reliability of the research data. Stool samples were outsourced to an external supplier for DNA extraction, quality control and preprocessing (see next section).

### 2.6. Bioinformatics

The wet-lab procedure, i.e. the physical, hands-on work to process and prepare samples from stool for genetic analysis, followed by the Bioinformatic Genetic Lab, Department of Internal Medicine Erasmus Medical Center, Rotterdam was the same in all cohorts (i.e. the same amplicon generated and the same sequencing). In OA2 and CON cohorts, stool DNA extraction was carried out using the DNA isolation kit Invimag Stool DNA Kit by Stratec and 100 mg of the stool sample. In OA1 the MagMax Microbiome Ultra kit from Thermo Fisher was used. The laboratory has tested the effect of the DNA isolation kit on bacterial profiles extensively and has concluded that the Ultra kit does not have significant deviation from the Invimag kit. In addition, in a direct comparison between the Invimag Stool DNA Kit and the MagMax Microbiome Ultra kit with other DNA extraction kits, these two kits performed comparably [50] and produced the best correlations with culture-based detection of specific pathogenic bacterial taxa [51]. In all cohorts, gut microbiome composition was determined by 16 S rRNA gene sequencing of the V3-V4 region carried out on an Illumina MiSeq, using V3 chemistry, at 2×300bp.

Quality control and preprocessing, including demultiplexing using QIIME1 version 1.9.1 and primer trimming with TagCleaner v0.16 was performed by the Bioinformatic Genetic Lab. Reads were exported from the MiSeq as a run level FASTQ file, containing all reads generated above Q30, the average QC value threshold Illumina uses. Above Q30 in short means that each nucleotide in the read has at maximum a 1:1000 chance of being called wrong. In OA1 and OA2, the DADA2 pipeline in R version 4.0.0 was used for analysis of the sequencing data that turns sample-wise amplicon sequences into a feature count [52]. ASVs (amplicon sequence variants) from DADA2 infer real sample sequence variants within a sample, and they are true, observed amplicon sequence fragments from samples. In other words, this software takes the genetic information from the samples and turns it into a count of different features. These features, called ASVs, help identify the unique genetic variations within each sample, giving us real and observed genetic fragments from the samples. In CON, read filtering and clustering was carried out using the MYcrobiota pipeline by SA. Briefly, chimeric sequences were filtered using the VSEARCH algorithm within Mothur, and reads were clustered into operational taxonomic units (OTUs) using closed-reference clustering against the SILVA database v132 based on a 97% similarity. In other words, similar genetic sequences were grouped together into categories known as OTUs based on their similarity to a reference database called SILVA v132, where they had to be at least 97% similar to be in the same group. Similarly for the DADA2 pipeline, the SILVA v132 was used as the reference database for taxonomic classification.

### 2.7. Statistical analysis

We carried out all statistical analyses in R v4.2.1. ASVs and OTUs with a relative abundance of < 0.01% in every sample were removed using a function adapted from Arumugam, Raes [53]. Zero and near zero variance ASVs and OTUs were also removed before further analyses using the caret package. Filtering resulted in 344 ASVs (from 1833) in OA1 and OA2 and 204 OTUs (from 2126) in CON remaining for final analysis. The filtered ASVs/OTUs were used for all downstream analyses. Results were only annotated at the genus taxonomic level. To account for the compositional nature of the microbiome data we applied a centred log-ratio (CLR) transformation before any statistical test. The Shannon index was calculated using the vegan package.

Differences between the descriptive characteristics of the three cohorts were evaluated using one-way ANOVA, Student *t*-tests or Mann–Whitney U tests for continuous variables, and Chi-squared test for categorical variables and Fisher’s exact test when < 5 in a category. OA1 served as the Discovery cohort and OA2 and CON as the Replication cohorts. We implemented a predictive algorithm in the Discovery cohort to identify a model or microbial signature containing the minimum number of features with the maximum predictive power. This algorithm is part of the *coda4microbiome* package and is designed to identify all possible pairwise log-ratios between pairs of components and perform variable selection through penalized regression on all the log-ratio pairs [28]. This analysis is comparing different taxa in pairs to identify if certain taxa tend to interact or coexist or if they compete with each other and testing which taxa pairs are the most relevant for explaining the trait of interest, in our case anxiety/depressive symptoms. The (weighted) balance between the taxa that contribute positively and negatively to the model comprises the microbial signature derived from this analysis, which tells us which taxa, when considered together, are particularly important for contributing positively and negatively to anxiety/depressive symptoms. Cross validation Mean Square Error (cv-MSE) is used to evaluate performance of the model, i.e. to make sure the analysis is accurate.

We fitted linear regression models to identify which of the taxa comprising the microbial signature were statistically significant and survived correction for age, sex, body mass index (BMI) and fibre intake in the Discovery cohort. We confirmed consistent directionality of our results in the two independent Replication cohorts and published literature. Linear regression results from each participating cohort were standardised to allow for the different scales. We performed z-score normalisation [54], also known as standardisation using the 'scale' function in R, and calculated Fisher's Z, commonly used to normalise correlations or effect sizes[55], based on the effect sizes from the published data (which did not alter the effect sizes reported in the original publications). Z-score normalisation involves subtracting the mean of the scores for each cohort from individual scores and then dividing by the standard deviation for that cohort. Fisher's Z transformation transforms correlation coefficients or effect size estimates into a distribution that is approximately normal, making it suitable for statistical analyses. In the final meta-analysis only the taxa that were significant in the Discovery cohort after adjusting for all covariates were included. However, we used the unadjusted standardised coefficients for all the cohorts when conducting the meta-analysis to ensure comparability between the cohorts and with Taylor et al. and Bosch et al., which adjusted for different or differently measured covariates. Meta-analysis takes the effect size, standard error and sample size into account to give an overall effect from the different cohorts studied. We used the R packages *metafor* and *meta* to perform both fixed-effects and Han-Eskin (H&E) random effect meta-analyses. The H&E random effect model was used in sensitivity analysis as it has higher statistical power than the fixed effects in the presence of heterogeneity [56]. Heterogeneity was measured with the I-squared *(I*^*2*^) and the Cochran's Q p-value. Publication bias was assessed visually using funnel plots and quantitatively with the Egger’s regression test and the trim- and fill-analysis [57], [58]. The p < .05 was considered statistically significant in all statistical analyses.

## 3. Results

### 3.1. Published literature: characteristics of the included studies

**Fig. 1:**
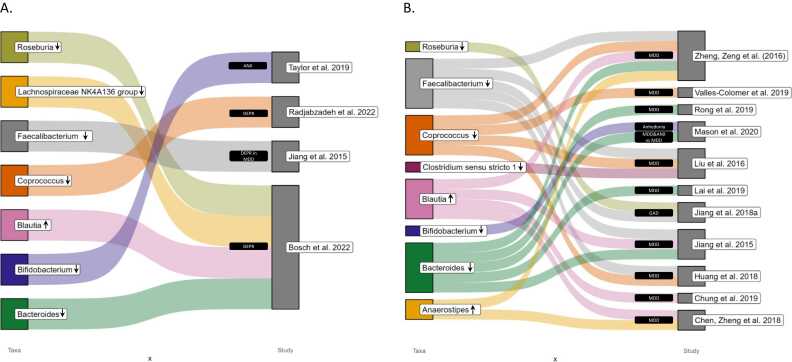
Sankey diagrams of selected taxa at the genus level related to anxiety or generalized anxiety disorder, depression or major depressive disorder, and anhedonia in previous literature with: A) taxa associated with higher or lower anxiety or depressive symptom scores in clinical and non-clinical populations and B) taxa whose abundances are significantly higher or lower in clinical populations with GAD/MDD/anhedonia and MDD and ANX, when compared to healthy controls and MDD only patients, respectively. MDD: Major depressive disorder. DEPR: depression/depressive symptoms. GAD: generalized anxiety disorder. ANX: anxiety or anxiety symptoms. ↑: higher abundance in patients or positive association with mood trait. ↓: Lower abundance in patients or negative association with mood traits.

A summary of the relevant results (i.e. associations between anxiety/depressive symptoms and the gut microbiota or comparisons of gut microbiota between cases and controls) from the 73 included studies is provided in Table S1, including taxa-specific analysis of genus and species level linked to anxiety or GAD, depression or MDD and other mood traits (e.g., anhedonia). A visual representation of selected genera is provided in Fig. 1**.**

#### 3.1.1. Published literature: methodological summary of included studies

Most studies employed 16 S rRNA gene sequencing to estimate gut microbiota composition (n = 64), two of which also included shotgun metagenomics. Six other studies used shotgun metagenomics to sequence all microbial genomes present in a sample. One study used oligonucleotide probes for *Bifidobacterium* and *Lactobacillus* species [59] and RT-qPCR was used by another study to quantify *Bifidobacterium* and *Lactobacillus* counts [60]. Metaproteomics analysis was performed by one study [61]. The majority of studies (n = 55) used a gold standard clinical interview or diagnostic criteria (e.g., DSM-IV/5, ICD-10th revision, Mini Neuropsychiatric Interview, Structured Clinical Interview for the DSM) to establish groups. Self-report measures of anxiety or depressed symptoms were utilised in fifteen studies. One study included two cohorts of people who were classified as having a depressive condition by their general practitioner or using a self-reported diagnosis [62]. A clinical cut-off score on the Hamilton Depression Rating Scale and self-report of an anxiety or depression diagnosis by a general practitioner were used in the two remaining trials to stratify cases and controls [63], [64].

#### 3.1.2. Study selection for quantitative synthesis

The majority of studies published to date dealt with clinical diagnoses of depression/MDD or GAD (n = 58). Systematic reviews with quantitative and qualitative syntheses for these traits have already been published elsewhere (e.g. [16], [17], [18]). Only 15 studies dealt with anxiety symptoms, depressive symptoms or both in a general population or community setting. The median sample size of these studies was n = 131, with larger median sample sizes for studies investigating depressive symptoms (n = 171) than anxiety symptoms (n = 49). The focus of the current research was to analyse the microbiome associations with depressive or anxiety symptoms.

For the quantitative synthesis in this study we have only included studies that reported regression coefficients with anxiety or depression scales in participants without a clinical diagnosis of mood disorders. The selection strategy is depicted in Fig. S1.

### 3.2. Consistency of published literature with data from three independent cohorts

The descriptive characteristics of the studied cohorts are shown in Table 1. There were no significant differences in demographic features, age, gender, or BMI, between the OA1 and OA2 cohorts. Differences in BMI were evident in both OA cohorts when compared to the CON group with the OA cohorts having higher BMI on average compared to CON. Moreover, we observed minor gender differences between the OA2 and CON groups, which we have addressed in the Discussion (Section 4). Knee pain severity was not measured in the control cohort but it might approximate 0 because participants reported that they did not experience knee pain. Anxiety and depressive symptoms did not differ in pairwise comparisons between the three groups. Higher Shannon diversity (richness/evenness) was observed in CON compared to both OA cohorts. Furthermore, within the OA cohorts, OA2 exhibited a higher Shannon diversity than OA1. However, Shannon diversity was not associated with anxiety or depressive symptoms in any of the three cohorts (data not shown).

**Table 1:** Descriptive characteristics of the three cohorts at baseline.

	OA1 (n = 46)	OA2 (n = 67)	CON (n = 58)	p-value (OA1 vs OA2)	p-value (OA1 vs CON)	p-value (OA2 vs CON)
	Mean ( ± SD)	Mean ( ± SD)	Mean ( ± SD)			
Age (y)	67.21 (6.01)	68.31 (7.58)	65.55 (9.32)	0.4238	0.3100	0.0704
	n = 43	n = 50				
Men/Women (%)	33/67	28/72	14/86	0.6569	0.0934	0.0439
	n = 43	n = 50				
BMI (kg/m^2^)	30.05 (6.42)	30.09 (5.66)	26.57 (4.51)	0.9752	0.0023	0.0005
	n = 39	n = 50				
Shannon mean	3.52 (0.74)	3.80 (0.65)	5.83 (0.86)	0.0357	< 0.0001	< 0.0001
Anxiety symptoms (%)	13.33^a^	6.67^b^	7.02^a^	0.3119	0.3314	> 0.9999
Depressive symptoms (%)	2.17^1^	8.00^2^	3.45^1^	0.3982	> 0.9999	0.4485
Knee Pain (NRS)	3.86 (2.34)	4.40 (1.99)	NA	0.1900	NA	NA
	n = 44	n = 47				
Opioids (%)	19.56	11.94	NA	0.2653	NA	NA
NSAIDs (%)	10.86	11.94	NA	0.8609	NA	NA

Abbreviations: BMI: body mass index; SD, standard deviation.

a. HADS-A & HADS-D: 0–7 = Normal, 8–10 = Borderline abnormal (borderline case), 11–21 = Abnormal (case); 13.33% and 7.02% had anxiety symptoms (≥11) and 2.17% and 3.45% experienced depressive symptoms in OA1 and CON, respectively.

b. A validated item from HADS-A & HADS-D subscale: 1–4, with 1 = Never to 4 = Always; 6.67% reported having anxiety symptoms and 8% reported having depressive symptoms in OA2.

OA1 served as the Discovery cohort, while the OA2 and CON cohorts served as Replication cohorts.

#### 3.2.1. Microbiome signatures predicting anxiety and depressive symptoms (Discovery cohort)

**Fig. 2:**
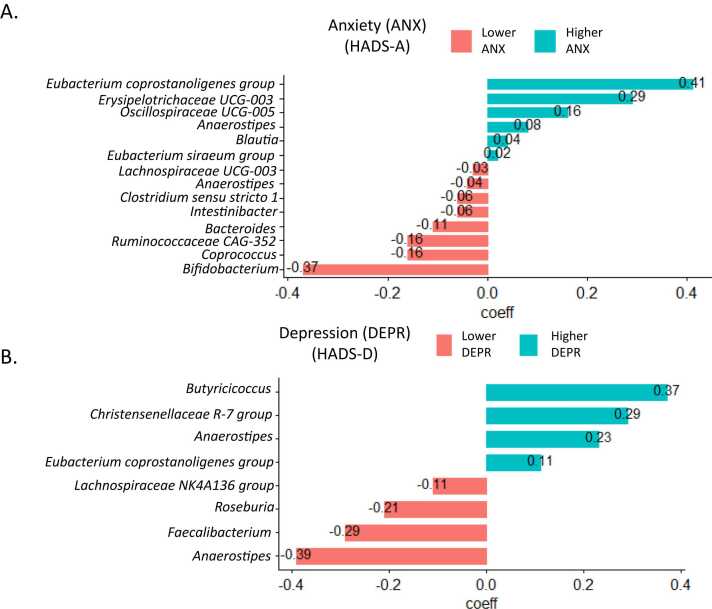
Microbial signature plots. Taxa at the genus level with their estimated regression coefficients that compose the signature that best predicts: A) anxiety (HADS-A) and B) depressive symptoms (HADS-D) in the Discovery cohort (n = 46). The magnitude of the coefficients represents the contribution of each variable to the model. Green: higher anxiety/depression (positive coefficient) and red: lower anxiety/depression (negative coefficient).

We first identified the microbial taxa that best predict anxiety and depressive symptoms in OA1 (Discovery cohort) using a prediction-based method (Fig. 2). The predictions plots that represent the association between the microbiome signature and the outcomes, and the cv-MSE curves for the two models are shown in Fig. S2. The mean cv-MSE is 9.75 [Standard Deviation (SD) = 1.9] and 8.04 (SD = 1.2) for anxiety and depressive symptom models, respectively.

#### 3.2.2. Associations of the signature taxa in all cohorts

We then conducted linear regressions with the aforementioned taxa, which are indicative of anxiety and depressive symptoms in the Discovery cohort, to examine their level of significance and determine whether they exhibit consistent directionality in the replication cohorts.

For anxiety symptoms, *Bifidobacterium, Clostridium sensu stricto 1, Coprococcus, Bacteroides, Blautia* and *Anaerostipes* (positive) were in the same direction in all cohorts (Table 2). *Bifidobacterium, Clostridium sensu stricto 1, Coprococcus* and *Bacteroides* had a negative association whereas *Blautia* and *Anaerostipes* (positive) had a positive association with higher anxiety symptom levels. As seen from Fig. 1 and Table S1 most of these taxa have been previously linked with depression. *Bifidobacterium* has been negatively associated with anxiety in females only in [42] and was found to be reduced in people with high anhedonia [25]. *Bacteroides* abundance was lower in people with comorbid MDD and anxiety relative to people with MDD alone [25]. The negative associations of *Bifidobacterium, Clostridium sensu stricto 1,* and *Coprococcus* with anxiety symptoms were significant only in the two OA cohorts.

**Table 2:** Linear regressions with anxiety and depressive symptom levels.

Anxiety symptoms	Discovery (OA1) (n = 46)			Replication (OA2) (n = 67)			Replication (CON) (n = 58)		
**Unadjusted**	**Beta**	**SE**	**p-value**	**Beta**	**SE**	**p-value**	**Beta**	**SE**	**p-value**
***Bifidobacterium***	**-0.45**	**0.14**	**0.001**	**-0.26**	**0.12**	**0.030**	**-0.004**	**0.13**	**0.973**
***Clostridium******sensu stricto******1***	**-0.36**	**0.14**	**0.013**	**-026**	**0.12**	**0.033**	**-0.03**	**0.13**	**0.821**
***Coprococcus***	**-0.35**	**0.14**	**0.018**	**-0.25**	**0.12**	**0.035**	**-0.19**	**0.13**	**0.173**
*Bacteroides*	-0.35	0.14	0.015	-0.12	0.12	0.335	-0.20	0.13	0.137
***Anaerostipes***	**0.34**	**0.14**	**0.022**	**0.13**	**0.12**	**0.277**	**0.21**	**0.13**	**0.132**
*Blautia*	0.30	0.14	0.041	0.13	0.12	0.299	0.17	0.13	0.202

**Depressive symptoms**									
*Butyricicoccus*	0.46	0.13	0.001	0.12	0.12	0.346	0.05	0.13	0.698
***Lachnospiraceae NK4A136 group***	**-0.38**	**0.14**	**0.008**	**-0.42**	**0.11**	**0.0003**	**-0.09**	**0.13**	**0.457**
*Roseburia*	-0.31	0.14	0.038	-0.20	0.12	0.096	-0.02	0.13	0.883

Taxa that were significant in the Discovery cohort and in the same direction in all three cohorts in unadjusted linear regression models. The taxa highlighted in bold remained significant in the Discovery cohort after adjusting for demographics (age and gender) and lifestyle (bodyweight (BMI) and dietary fibre intake) covariates. Abbreviations: SE, standard error. *p < 0.05, * *p < 0.01.

For depressive symptoms, *Lachnospiraceae NK4A136 group*, *Butyricicoccus* and *Roseburia* were in the same direction in all cohorts (Table 2). *Lachnospiraceae NK4A136 group* and *Roseburia* taxa had a negative association, whereas *Butyricicoccus* had a positive association with higher depressive symptom levels. Similarly, Bosch et al. (2022) also found a negative association of *Lachnospiraceae NK4A136 group* and *Roseburia* taxa with higher depressive symptom levels [43]. Inconsistent findings have been previously reported for *Butyricicoccus.* For example, Chen, et al. [65] reported increased abundance of the genus *Butyricicoccus* in controls relative to MDD, while Jiang and co-workers [23] found a decreased abundance of this genus in GAD compared to controls.

The rest of the taxa identified in 3.2.1 that comprised the signatures predicting anxiety and depressive symptoms were not found to be in the same direction in all three cohorts (data not shown). For instance, *Faecalibacterium* taxon had a negative association with higher depressive symptom levels in OA1 but was found to be in the opposite direction in OA2 and CON. In the majority of studies *Faecalibacterium* taxon had lower abundance in depression (e.g., [66], [67], [68]) consistent with the OA1 cohort except for one study which found higher abundance of this taxon in MDD in the females only group [64].

Linear regressions with the taxa that comprised the signatures predicting anxiety and depressive symptoms were adjusted for the a priori selected covariates (age, sex, BMI and dietary fibre intake) in the Discovery cohort. The taxa that remained statistically significant after adjustment for demographic and lifestyle covariates are highlighted in bold in Table 2. The percentage mean relative abundances of the signature taxa can be found in Table S3 (Supplementary material). For anxiety, the majority of taxa remained significant after adjustment apart from *Bacteroides* and *Blautia*. Out of those, *Bifidobacterium* was the only genus previously found to be linked to anxiety symptoms in Taylor, Thompson [42] among females (Fig. 1). For depressive symptoms, out of the three taxa only *Lachnospiraceae NK4A136 group* remained significant in the Discovery cohort after adjusting for covariates and has been previously linked to depressive symptoms in Bosch, Nieuwdorp [43]. As seen from Table 2, *Bifidobacterium* and *Lachnospiraceae NK4A136 group* taxa were negatively associated with anxiety and depressive symptom levels in all three cohorts but remained significant only in the two osteoarthritis cohorts.

#### 3.2.3. Meta-analysis of de novo data and published literature

**Fig. 3:**
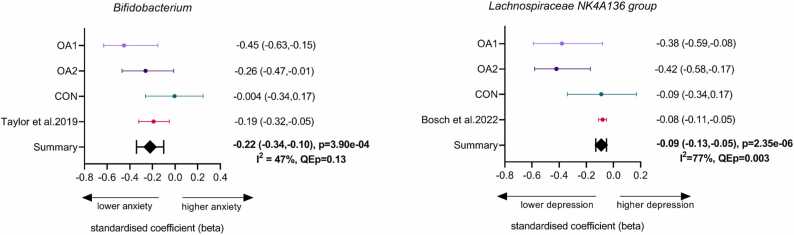
Forest plots of the meta-analysis results from three independent cohorts (OA1, OA2, and CON) and published literature (Taylor et al., 2019 and Bosch et al., 2022) for: A. anxiety symptoms and *Bifidobacterium* and B. depression symptoms and *Lachnospiraceae NK4A136*. I^2^ (I-squared) and QEp (Cochran's Q P value) were used to measure heterogeneity.

The two genera that remained significant after adjustment for covariates and were previously reported in the literature to be associated with anxiety and depression symptoms were *Bifidobacterium* and *Lachnospiraceae NK4A136 group*, respectively. We conducted meta-analyses with the data from our own cohorts and published studies to estimate the pooled effect of those genera in relation to anxiety and depressive symptoms (Fig. 3). *Bifidobacterium* was reproducibly associated with anxiety symptoms in each of the three cohorts and Taylor, Thompson [42] [fixed-effect beta across the cohorts (95% CI) = −0.22 (−0.34, −0.10), p = 3.90e-04]. *Lachnospiraceae NK4A136 group* was consistently associated with depressive symptoms in each of the three cohorts and Bosch, Nieuwdorp [43] [fixed-effect beta across the cohorts (95% CI) = −0.09 (−0.13, −0.05), p = 2.53e-06]. We did not observe significant heterogeneity in the meta-analysis of *Bifidobacterium* taxon with anxiety symptom scores (QEp > 0.05). There was significant heterogeneity in the meta-analysis of *Lachnospiraceae NK4A136 group* taxon with depressive symptom scores (QEp < 0.05), which we followed up by conducting sensitivity analyses (see next section). The funnel plot for *Bifidobacterium* showed no evidence of publication bias (i.e., symmetry on inspection of the plot), whereas the *Lachnospiraceae NK4A136 group* plot indicated potential publication bias as there was some asymmetry (Fig. S3). However, the Egger’s test was not significant for either *Bifidobacterium* or *Lachnospiraceae NK4A136 group* (p = 0.77 and 0.20, respectively), indicating no evidence of publication bias. In addition, the trim- and fill- analysis produced almost identical results compared to our original meta-analyses [*Bifidobacterium* estimate (95% CI) = −0.22 (−0.34, −0.10), p = 4.0e-04, and *Lachnospiraceae NK4A136 group* estimate (95% CI) = −0.08 (−0.12, −0.04), p < .0001], further supporting a lack of publication bias. A potential source of asymmetry in the funnel plot for the meta-analysis of *Lachnospiraceae NK4A136 group* with depressive symptom levels may be the presence of heterogeneity.

#### 3.2.4. Sensitivity analyses

We examined whether the results differed if OA2 was excluded from the meta-analysis due to the different instruments used to measure anxiety and depression symptoms in this cohort; the two validated items from the HADS as opposed to the full HADS used in OA1 and CON. Leave-one-out meta-analysis including only OA1 and CON resulted in the bacterial taxa *Bifidobacterium* and *Lachnospiraceae NK4A136 group* to remain statistically significant with negative associations with anxiety and depressive symptoms [fixed-effect beta (95% CI) = −0.20 (−0.34, −0.06), p = 4.98e-03, QEp = 0.06, and fixed-effect beta (95% CI) = −0.08 (−0.12, −0.04), p = 1.69e-05, QEp = 0.10], respectively.

Considering the heterogeneity between the various cohorts, both ours and in the literature (Fig. 3), we also ran a H&E random-effects meta-analysis. The results showed that both *Bifidobacterium* and *Lachnospiraceae NK4A136* taxa remained significantly negatively correlated with anxiety and depressive symptoms [HE random-effect beta (95% CI) = −0.22 (−0.36, −0.07), p = 3.9e-3, QEp = 0.13; HE random-effect beta (95% CI) = −0.22 (−0.40, −0.04), p = 1.76e-2, QEp < .01], respectively. Nevertheless, heterogeneity remained significant in the meta-analysis with *Lachnospiraceae NK4A136* taxon.

Two different pipelines were used for microbiome analysis; DADA2 was used in the two osteoarthritis cohorts and MYcrobiota in the CON cohort. To check for potential incompatibilities between the two approaches, we compared the standardised beta estimates of the *Bifidobacterium* and *Lachnospiraceae NK4A136* taxa associations in a small subset of the OA2 cohort for which we had microbiome data analysed with both pipelines (n = 33). Regardless of the pipeline, *Bifidobacterium* and *Lachnospiraceae NK4A136* taxa were negatively associated with anxiety and depressive scores, respectively (*Bifidobacterium*: DADA2 beta = −0.29 and MYcrobiome beta = −0.12; *Lachnospiraceae NK4A136*: DADA2 beta = −0.59 and MYcrobiome beta = −0.13). Nevertheless, we observed differences in the magnitude of the effects between the two pipelines with DADA2 producing greater effects, especially for *Lachnospiraceae NK4A136* taxon. This is consistent with the observation that some of the findings were only significant in the two osteoarthritis cohorts and not in the CON cohort, therefore indicating some level of indirect incompatibility between the two approaches. Despite this, our findings are valid as we could replicate them across our de novo data and published literature with the overall effect remaining significant, including in the random-effects models.

Finally, because our study used two chronic pain cohorts we also tested the potential effect of opioids and nonsteroidal anti-inflammatory inhibitors (NSAIDs) in our own datasets conducting a subanalysis of the linear regressions with anxiety and depressive symptom levels adjusting for use of pain medications (NSAIDs and opioids) in both OA cohorts. The results of this supplementary analysis, presented as Table S2 (Supplementary material), demonstrate that all our findings remain statistically significant even after accounting for use of analgesics. Importantly, the effect sizes in these adjusted models exhibit minimal variations. For example, the effect size of *Bifidobacterium* increased from − 0.45 to − 0.47 after adjusting for medication use whereas the effect size of *Lachnospiraceae NK4A136 group* decreased from − 0.38 to − 0.33 after adjustment. Neither NSAID nor opioid use was significantly associated with prevalence of anxiety or depressive symptoms in the two chronic pain cohorts tested.

## 4. Discussion

In this study we have for the first time carried out a meta-analysis of microbiome features associated with anxiety and depressive symptoms using both new data and data from the literature. We find consistent and robust associations between the presence of the *Bifidobacterium* genus and lower anxiety symptom levels. We also find a consistent association between *Lachnospiraceae NK4A136 group* and lower depressive symptom levels also reported in the HELIUS cohort by Bosch et al. Heterogeneity between cohorts was observed in the meta-analysis with *Lachnospiraceae NK4A136* taxon, which suggests the need for further studies to confirm its actual effect.

No significant associations in richness/evenness measured with the Shannon diversity index were observed with either anxiety or depressive symptoms across the three cohorts. This is consistent with evidence from a meta-analysis in MDD [15] and with our systematic search of the literature which found more studies that reported no significant associations with Shannon or other measures of alpha diversity in anxiety/depression (n = 40), compared to studies that found lower alpha diversity across all measured indices (n = 16). It is possible that taxon- specific microbial features might play a more prominent role in depression, rather than variations in global compositional features. In order to elucidate the influence of alpha diversity on anxiety symptoms more studies are required.

One aim of the current study was to explore potential differences in microbial features previously associated with anxiety or depressive symptoms among individuals with or without chronic pain. A notable finding is that the magnitude and significance of the associations between several microbial taxa (i.e. *Bifidobacterium*, *Clostridium sensu stricto 1* , *Coprococcus* and *Lachnospiraceae NK4A136 group*) and anxiety/depressive symptoms were stronger in the two osteoarthritis cohorts than in the CON group in our de novo data. While this effect could partly be driven by the different pipelines used for microbiome analysis as evident from the sensitivity analyses, we cannot disregard the possibility that some of the variation may be driven by the presence of chronic pain. Consistent with this notion are reports that *Bifidobacterium* and *Clostridium* genera are also reduced in fibromyalgia patients [33]. There is a biological rationale for these observations, given the strong link between the pathways underlying pain, anxiety and depression [69], [70]. A recent study [71] showed that anxiety enhances pain in patients with OA likely through an enhanced activation of astrocytes in the periaqueductal gray and anterior cingulate cortex brain regions that have well-documented roles both in anxiety [72] and osteoarthritis pain [73], [74]. *Bifidobacterium* may therefore have a potential pleiotropic effect influencing the pathways shared by anxiety and chronic pain.

We also investigated the role of long-term use of pain medications such as opioids and NSAIDs given that these could potentially be responsible for the gut microbiome associations observed [75], [76]. When we adjusted for use of analgesics, the effect sizes were essentially the same as without the adjustment and notably, neither NSAID nor opioid use was associated with prevalence of anxiety or depressive symptoms in our in house cohorts. Our results are consistent with the finding from a recent two-sample bi-directional Mendelian randomization study which found no evidence of significant causal association between prescription opioid usage and host genetic-driven gut microbiome composition [77].

After full adjustment for covariates (age, sex, BMI and fibre intake) in the Discovery cohort only the association between the presence of *Lachnospiraceae NK4A136 group* and lower levels of depressive symptom remained significant. However, in the meta-analysis with Bosch, Nieuwdorp [43] this taxon exhibited significant heterogeneity, suggesting that more studies are needed to gain a better understanding of its effect. A recent systematic review found that the family *Lachnospiraceae* had consistently lower relative abundance (in 4/6 studies) in individuals with depression compared to controls [12]. In our systematic search of the literature, we found three studies reporting a negative association or an increase in abundance of *Lachnospiraceae NK4A136 group* in controls versus MDD [43], [65], [78] and two trials that found a decrease in the abundance of *Lachnospiraceae NK4A136 group* in GAD [20], [21]*. Lachnospiraceae NK4A136 group* therefore seems to exert beneficial effects on negative affect in general rather than having a specific association with either anxiety or depressive disorders and symptoms. This is in line with the idea that changes in the gut microbiome may be associated with overall changes in psychological health instead of showing taxonomic specificity, whereby specific taxa differentiate specific disorders.

The consistent association between the presence of the *Bifidobacterium* genus and lower levels of anxiety symptoms across our three cohorts and in the published literature withstood adjustments for heterogeneity and fibre intake both in our Discovery cohort and in Taylor, Thompson [42]. Understanding the links between the gut microbiome and anxiety and depression provides a fresh perspective in mental health research and potential for developing nutritional therapies. It is well known that prebiotic fibre interventions, such as inulin, result in consistent increases in *Bifidobacterium* [79]. In addition, *Bifidobacterium* strains are among the most widely used probiotics [80]. A meta-analysis found that probiotics attenuated both depression and anxiety although with small effects [81] due to the literature in humans being sparse, heterogeneous and with significant limitations [81], [82]. Another meta-analysis failed to find an association with depression [83]. It must be noted that this genus is highly diverse [84] and therefore the inconsistency of previously reported results could be simply due to the administration of different highly strain-specific supplements.

The beneficial effect of *Bifidobacterium* genus reported here could be explained by its ability to produce acetate and lactate during carbohydrate fermentation, which in turn are converted into butyrate by butyrate-producing bacteria through cross-feeding interactions [84]. Interestingly, a recent metabolomic study found that reduced levels of plasma lactate were linked to a history of susceptibility to MDD rather than lifetime resilience to MDD, and predicted future susceptibility to MDD regardless of socioeconomic factors and physical disease burden [85]. Furthermore, an anti-inflammatory potential of *Bifidobacterium* has been demonstrated in an animal model [86] and probiotics containing *Bifidobacterium spp*. have been shown to attenuate stress response in mice by modulating hippocampal neurogenesis and synaptic plasticity [87]. *Bifidobacterium infantis* species in particular was shown to reverse the stress response by inducing c-Fos activation in the paraventricular nucleus in germ-free mice [88]. Similarly, genera of the *Lachnospiraceae* family are integral components of the gut microbiota and are prominent producers of short-chain fatty acids [89], which is likely the underlying cause of their beneficial impact in depression or depressive symptoms as we and others demonstrated.

By identifying the key bacterial candidates associated with anxiety and MDD and stress resilience-promoting microorganisms like *Bifidobacterium*, personalized nutritional therapies can be developed to target specific bacteria and their interactions with the gut-brain axis. Miyaoka et al. have observed that the combination of antidepressants and probiotics is more effective to treat drug-resistant depression [90] which may be a viable adjuvant treatment option for patients with depression. In addition, targeted probiotics interventions could be used to aid in the prevention of new cases of MDD and of relapses due to stress in depressed patients.

Strengths of the current study include an updated and extensive systematic literature search both for depression and anxiety and the inclusion of dietary fibre as covariate in the Discovery cohort. The present analysis on the microbial signatures was based specifically on model prediction rather than differential abundance in the Discovery cohort. Another strength is the cross-validation using three databases and performing meta-analysis with previous published literature and the use of both chronic pain and non-chronic pain cohorts which has enabled us to observe potential differences in microbiome associations with anxiety and depression in these two groups. The results from our de novo data analysis are based on adults without clinical depression, anxiety, or stress disorders, and therefore can be extended to the general population that experiences negative mood states at a subclinical level.

We note some study limitations. Our aim was to investigate the link between gut microbiome and anxiety/depressive symptom levels across their full spectrum at a subclinical level in the general adult population and not only in individuals at the extreme end of the spectrum such as those with a diagnosed psychiatric illness. Therefore, our results may not translate to individuals with a clinical diagnosis of anxiety or depression. Nevertheless, we used validated instruments to measure anxiety and depression levels derived from a widely used scale (HADS) that are highly correlated with clinical screening [91]. We also note the use of two validated single questionnaire items from HADS-A and HADS-D sub-scales for measuring anxiety and depression levels, respectively, in one of the chronic pain cohorts (OA2) and not the full scale that was used in the other two cohorts (OA1 and CON). These items are highly correlated with the full HADS-A and HADS-D and showed the highest significant (p < 0.05) associations with latent traits of anxiety and depression, respectively, in exploratory structural modelling analysis carried out by Akin-Akinyosoye, Frowd [92]. Nevertheless, they are potentially less sensitive than HADS-A for anxiety symptoms and more sensitive than HADS-D for depressive symptoms (Table 1). Because of the likely noise that this could introduce, we conducted a leave-one-out meta-analysis which provided very similar results. Another limitation is that the OA1 cohort does not express high levels of depression making it less likely to be ideal for the discovery of novel bacterial taxa associated with depressive symptoms. Despite this, we find that several of the taxa identified from the prediction-based analysis in OA1 (Fig. 2), such as the *Lachnospiraceae NK4A136 group*, *Roseburia*, and *Faecalibacterium*, have been identified in the literature (Fig. 1) to be associated with depressive symptoms or to change in depressed patients compared to controls in the same direction. This supports our study's goal of validating and highlighting consistent and robust associations based on those already reported in the literature. Another methodological difference is the use of ASVs in all the cohorts (i.e. OA1, OA2, Taylor et al. and Bosch et al.) except for CON which used OTUs. Sensitivity analyses in the subset of data from OA2 for which we had both OTUs and ASVs resulted in the same direction of effect for *Bifidobacterium* and *Lachnospiraceae NK4A136 group*, and reproduced associations were observed for the two taxa, suggesting robust associations of anxiety and depressive symptoms, respectively. We acknowledge that despite evidence from the literature that the two DNA extraction kits are comparable we cannot exclude the possibility that the two different kits might have a slight deviation on a per profile basis but this is expected to be very minor as compared to other sources, such as biological variation or gene target region variation [93], [94].

Another potential limitation may be seen in the marginal gender differences between the OA2 and CON groups. The higher proportion of women in the control group result of this having started as a female only cohort although it is widely recognised as representative of the general female UK population [95]. Although this might have introduced some bias we only included in the meta-analyses the genera that remained significant after adjusting for age, gender, BMI and fiber intake in the OA1 cohort. In addition, in both OA2 and CON adjusting for gender the associations of anxiety and depressive symptoms with *Bifidobacterium* and *Lachnospiraceae NK4A136 group*, respectively, has only minor effects in the standardized effect sizes in either cohort [OA2: *Bifidobacterium* effect size= −0.29 (SD=0.15) and *Lachnospiraceae NK4A136 group* effect size= −0.43 (SD=0.12); CON: *Bifidobacterium* effect size= −0.009 (SD=0.13) and *Lachnospiraceae NK4A136 group* effect size= −0.09 (SD=0.13)]. Therefore, we do not expect these minimal gender differences to impact our results.

In conclusion, we report that two bacterial genera, *Bifidobacterium* and *Lachnospiraceae NK4A136 group* are reproducibly associated with lower anxiety and depressive symptoms, respectively. Heterogeneity between cohorts was observed only for the association with *Lachnospiraceae NK4A136 group*, suggesting that more meta-analyses are needed to elucidate its effect. The association with *Bifidobacterium* was not affected by heterogeneity and supports previous observations of its potential prophylactic effect against anxiety symptoms.

## Funding

This study was supported by grant the NIHR Nottingham Biomedical Research Centre, by Versus Arthritis Project grants 22473 and 23139, and by 10.13039/100014013UKRI grant MR/W026813/1. The control cohort was funded by the 10.13039/100011721Chronic Disease Research Foundation. TwinsUK receives funding from the 10.13039/100004440Wellcome Trust, the 10.13039/501100000780European Commission H2020 grants SYSCID (contract #733100); the 10.13039/501100000272National Institute for Health Research (NIHR) Clinical Research Facility and the 10.13039/100014461Biomedical Research Centre based at Guy's and St Thomas' NHS Foundation Trust in partnership with King's College London, the 10.13039/100011721Chronic Disease Research Foundation, the UKRI Medical Research Council (MRC)/British Heart Foundation Ancestry and Biological Informative Markers for Stratification of Hypertension (AIM-HY; MR/M016560/1), and Zoe Limited. This work was also supported by UKRI grant MR/W026813/1 to AMV and CM is supported by the 10.13039/100011721Chronic Disease Research Foundation.

## CRediT authorship contribution statement

**Afroditi Kouraki:** Conceptualization, Methodology, Software, Formal analysis, Data curation, Writing − original draft, Writing − review & editing, Visualization. **Anthony Kelly:** Investigation, Resources, Data curation, Writing − review & editing, Project administration. **Amrita Vijay:** Investigation, Resources, Data curation, Writing − review & editing, Project administration. **Sameer Gohir:** Investigation, Resources, Data curation, Writing − review & editing, Project administration. **Stuart Astbury:** Software, Formal analysis, Writing − review & editing. **Vasileios Georgopoulos:** Investigation, Writing − review & editing. **Bonnie Millar:** Project administration, Data curation, Writing − review & editing. **David Andrew Walsh:** Resources, Funding acquisition, Writing − review & editing. **Eamonn Ferguson:** Methodology, Writing − review & editing. **Cristina Menni:** Methodology, Resources, Writing − review & editing, Funding acquisition. **Ana M Valdes:** Conceptualization, Methodology, Supervision, Writing − original draft, Writing − review & editing, Funding acquisition.

## Declaration of Competing Interest

AMV and AV are members of the Chuckling Goat (a nutrition company) Scientific Advisory Board. AMV is a member of the CP Kelco SAB. AMV and CM are consultants for Zoe Ltd. DAW has undertaken consultancy through University of Nottingham for Contura International A/S, Glaxo SmithKline, AKL Research and Development Ltd, Pfizer Ltd, Abbvie Ltd, Ely Lilly & Co.Ltd, Galapagos Ltd., and Reckitt Benckiser Health Ltd and is responsible for research grants paid to University of Nottingham from Versus Arthritis, UKRI, NIHR, Pfizer Ltd, Ely Lilly & Co Ltd, UCB Pharma, and Orion Pharma (all non-personal pecuniary interests).
