# Supplementary material for: Reproducible microbiome composition signatures of anxiety and depressive symptoms
Source: Comput Struct Biotechnol J. 2023 Oct 18;21:5326–36. doi: 10.1016/j.csbj.2023.10.035 (PMC10637863; doi:10.1016/j.csbj.2023.10.035)
Supplement: Supplementary file 1 — Supplementary material [file mmc1.docx]

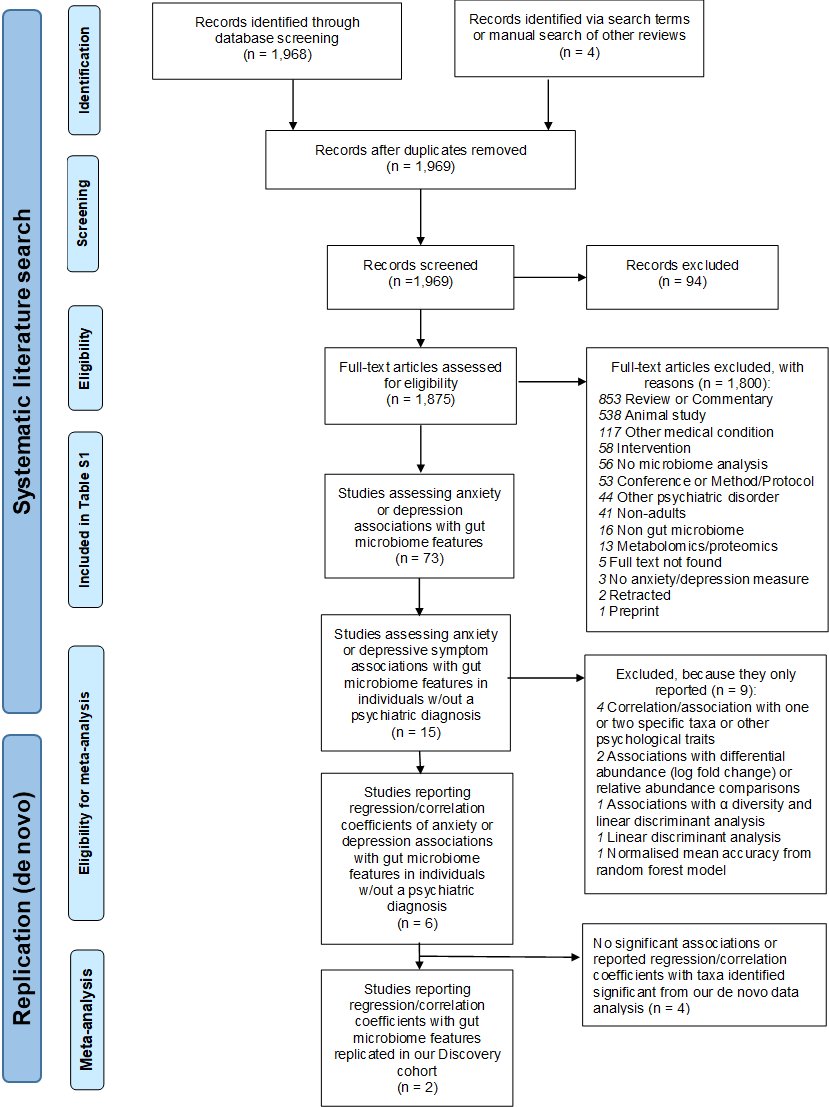


**Figure S1.** CONSORT diagram

**Table S1.** Summary of studies reporting anxiety or depression associations with gut microbiome features.

| **Study with groups and sample size** | **Sequencing** | **Anxiety/depression measures** | **Taxonomic differences** | | | **Alpha & Beta diversity/Other** |
| --- | --- | --- | --- | --- | --- | --- |
| **1) Aizawa et al. (2016)** Case-control, n=100  (MDD, n=43; Controls, n=57)  DOI:10.1016/j.jad.2016.05.038 | Bacteria:  RT-qPCR for Bifidobacterium and Lactobacillus counts | Diagnosis:  Psychiatrist performed utilising DSM-IV  Symptoms:  Hamilton Depression Rating Scale (21-item version) | **qPCR for *Bifidobacterium* and *Lactobacillus***  ↓ *Bifidobacterium* counts in MDD When males and females were analysed separately this was only significant for males | | | **Alpha and beta diversity**  Did not analyse |
| **2) Bai et al. (2021)**  Case-control, n=120  (MDD, n=60; controls, n=60)  DOI: 10.2147/JIR.S324922 | Bacteria:  16S rRNA gene sequencing | Diagnosis: Psychiatrist performed utilising DSM-IV  Symptoms:  Hamilton Depression Rating Scale | 17 differential genera responsible for the discrimination between the two groups: Lactobacillus, Colidextribacter, Holdemania, norank_f_Lachnospiraceae, GCA-900066575, Lachnoclostridium, Eubacterium_ventriosum_group, Candidatus_Soleaferrea, Faecalitalea, CAG-56, Lachnospiraceae_NK4A136_group, Lachnospira, Clostridium_innocuum_group, unclassified_f_Lachnospiraceae, Collinsella, Parabacteroides, and Klebsiella. The former 14 differential genera belonged to phyla Firmicutes. | | | **Alpha and beta diversity**  No significant differences in Shannon, Chao, Simpson and Phylogenetic diversity  Significant difference in beta-diversity (PCoA) |
| **3) Bai et al. (2022)**  Case-control, n=112  (MDD, n=56; matched controls, n=56)  DOI: 10.3389/fcimb.2022.831186 | Bacteria:  16S rRNA gene sequencing - Illumina MiSeq platform | Diagnosis: Structured Psychiatric Interview using DSM-IV-TR criteria  Symptoms:  Hamilton Depression Rating Scale | ↓ 8 OTUs in MDD  ↑ 38 OTUs in MDD  28 OTUs belonging to Firmicutes (8 OTUs belonging to family Lachnospiraceae, 6 OTUs belonging to family Oscillospiraceae and 6 OTUs belonging to family Ruminococcaceae) and 11 OTUs belonging to Bacteroidota (4 OTUs belonging to family Bacteroidaceae and 3 OTUs belonging to family Rikenellaceae)  9/14 OTUs belonged to Firmicutes, and five to family Lachnospiraceae under Firmicutes | | | **Alpha and beta diversity**  No significant differences in Richness (Chao1) and Richness/evenness (Shannon)  Difference in beta-diversity (PLS-DA)  **Other**  14 differential OTUs significantly correlated with HDRS |
| **4) Bosch et al. (2022)**  Cross-sectional associations between depressive symptoms and gut microbiota across 6 ethnic groups**,** n = 3211  DOI: 10.1038/s41467-022-34504-1 | Bacteria:  16S rRNA gene sequencing - Illumina MiSeq platform | Symptoms:  9-item Patient Health Questionnaire-9 | 70 taxa remained significantly associated with PHQ-9 scores after adjustment for age, gender and ethnicity. 60/70 ASVs belonged to the phylum Firmicutes, with a prominent presence of the genus Christensenellaceae (group R7) and various genera within the families Lachnospiraceae (e.g., Blautia, Lachnospiraceae NK4A136, Marvinbryantia, Roseburia) and Ruminococcaceae (e.g., Oscillibacter, Ruminicoccus 1, Ruminococcaceae NK4A214 group, Ruminococcaceae UCG-005). Less prominent phyla included Bacteroidetes (e.g., genus Bacteroides) and Proteobacteria (genus Desulfovibrio and Escherichia/Shigella)  -ASVs within the same genus showed opposite associations with depressive symptom scores, e.g., Blautia, Bacteroides, and Oscillospira  -other genera a more consistent pattern of associations was observed (e.g., Christensenellaceae, Desulvofibrio, Streptococcus)  -ASVs identifying the genus Bifidobacterium showed a positive correlation with depression | | | **Alpha and beta diversity**  Diversity within (alpha-diversity) and between individuals (beta-diversity) predicts depressive symptom levels, taking into account demographic, behavioural, and medical differences; these associations do not differ between ethnic groups  Beta-diversity explains 29%–18% of the ethnic differences in depressive symptoms |
| **5) Butler et al. (2023)**  Case-control, n=49 (SAD, n=31; Matched controls, n=18)  DOI: 10.1038/s41398-023-02325-5 | Whole genome shotgun sequencing | Diagnosis:  psychiatrist using the MINI International Neuropsychiatric Interview and DSM-V  Symptoms: Liebowitz Social Anxiety Scale-Self Report | **Significant differences in relative abundance after FDR correction:**  -*Genus level*  ↑ Anaeromassilibacillus &Gordonibacter in SAD  ↑ Parasutterella in controls  *-Species level*  ↑ Anaeromassilibacillus sp An250  ↑ Parasutterella excrementihominis in controls | | | **Alpha and beta diversity**  No differences in alpha diversity (Chao1, Shannon or Simpson indices) ​  Beta diversity was significantly different between the two groups (Aitchison distance metric)  **Other (functional differences)**  ↑ Aspartate Degradation I in SAD |
| **6) Caso et al. (2021)**  Case-control, n=113  (Active-MDD, n=46;  Remission or mild symptoms MDD, n=22; Controls, n=45)  DOI: 10.1038/s41398-021-01755-3 | Bacteria:  16S rRNA gene sequencing - Illumina | Diagnosis:  structured clinical interviews using DSM-IV-TR  Symptoms:  HDRS (Spanish version),  Euroquol-5D visual analog scale, and others | **Metataxonomic analysis**  -*Genus level*  ↑Bilophila and Alistipes in MDD (both groups combined)  ↓Anaerostipes and Dialister in MDD  ↑ Alistipes in active-MDD  ↑Bilophila in remission-MDD | | | **Alpha and beta diversity**  Diversity (Shannon) was not different among the groups  No difference in beta diversity (distance matrices with Bray–Curtis dissimilarity and binary Jaccard distance, PCoA)  **Other**  Differences in inflammatory markers |
| **7) Chahwan et al. (2019)** Case-control, n=91  (Clinical/  Subclinical Depression, n = 71 [n = 68 provided stool samples]; Controls, n=20)  DOI: 10.1016/j.jad.2019.04.097 | Bacteria:  16S rRNA gene sequencing - Illumina MiSeq platform | Diagnosis: Clinician administered MINI Symptoms: DASS-21 Beck Depression Inventory - II Beck Anxiety Inventory Leiden Index of Depression Sensitivity-Revised | *Ruminococcus gnavus* was positively correlated with DASS depression scores, and was found in 72% of depressed participants compared to 25% in the healthy controls. It was present in higher relative abundance in the severe BDI range of depression (BDI > 28, *n* = 36) in comparison to both the mild/moderate depressed range (BDI = 12- 28, *n* = 31) and healthy controls (BDI ≤ 10).  No significant difference in relative abundance between depression and control groups at any taxonomic level. | | | **Alpha and beta diversity**  No significant differences in Richness (number of OTUs, Chao1) and Richness/evenness (Shannon)  No significant difference bet groups on PCoA/PERMANOVA (weighted UniFrac) |
| **8) Chen, Chung et al. (2022)**  Case-control, n=20 (MDD, n=10; Controls, n=10)  DOI: 10.1038/s41598-022-24773-7 | Bacteria:  16S rRNA gene sequencing - Illumina MiSeq platform | Diagnosis:  Psychiatrics diagnosis with DSM-V  Symptoms:  Beck Depression Inventory, Beck Anxiety Inventory,  Perceived Stress Scale | *-Phylum level*  ↑ Bacteroidetes in MDD  ↓ Actinobacteria in MDD  *-Genus level*  Differential expression of 270 Genera revealed 27 that had differential abundance (fold change) >1 and only the following 7 were presented in over 90% of all faecal samples:  Anaerostipes, Bacteroides, Bifidobacterium, Clostridium, Collinsella, Dialister, and Roseburia  ↑ Bacteroides in MDD  ↓ Anaerostipes, Bifidobacterium, Clostridium, Collinsella, Dialister, and Roseburia in MDD | | | **Alpha and beta diversity**  Both richness and the Shannon diversity index did not show significant differences bet the groups |
| **9) Chen, He et al. (2020)**  Case-control, n=141 [Age-specific differences in MDD versus controls (Young group MDD n=25; vs Control, n=27 and Middle-aged  MDD n=45; vs Control, n=44]  DOI: 10.18632/aging.102775 | Bacteria:  16s rRNA gene sequencing | Diagnosis:  Psychiatrists-based using the DSM-IV-based Composite International Diagnostic Interview  Symptoms:  Hamilton Depression Rating Scale | **Young**  **Differential OTUs**  **-Random forest algorithm**  92 OTUs responsible for the separation between young MDD patients and young controls  Families: Bacteroidaceae, Clostridiaceae_1, Coriobacteriaceae, Erysipelotrichaceae, Lachnospiraceae, Peptostreptococcaceae and Ruminococcaceae  **-LEfSe**  *-Family level*  ↑ Clostridiaceae_1, Peptostreptococcaceae and Ruminococcaceae in controls  *-Genus level*  ↑Clostridium_sensu_stricto, Clostridium_XI, and Clostridium_XVIII in controls | **Middle Aged**  **Differential OTUs**  **-Random forest algorithm**  122 OTUs responsible for the separation between middle-aged MDD patients and middle-aged controls  Families: Lachnospiraceae, Coriobacteriaceae, Streptococcaceae, Prevotellaceae, Bacteroidaontrolsceae, Eubacteriaceae, Actinomycetaceae, Sutterellaceae, Acidaminococcaceae, Erysipelotrichaceae, Ruminococcaceae, and Porphyromonadaceae  **-LEfSe**  *-Family level*  ↑ Lachnospiraceae, Coriobacteriaceae, Streptococcaceae, Eubacteriaceae, Actinomycetaceae in MDD  ↑ Sutterellaceae, Acidaminococcaceae, Prevotellaceae, Bacteroidaceae in controls  *-Genus level*  ↑ Clostridium_XVIII, Anaerostipes, Veillonella, Streptococcus, Eggerthella, Roseburia, Eubacterium, Actinomyces in MDD  ↑ Anaerovorax, Sutterella, Oscilibacter, Phascolarctobacterium, Coprococcus, Bacteroides, Feacalibacterium in controls | | **Alpha and beta diversity**  No significant differences in ACE and Chao1 indexes in either group (young or middle aged)  Differences in microbial abundance in both groups with OPLS-DA (young MDD versus young controls and middle aged MDD vs middle aged controls)-no statistical test |
| **10) Chen, Li, et al. (2018)** Case-control, n=20 (MDD, n=10; Controls, n=10)  DOI: 10.1097/WNR.0000000000000985 | Metaproteomics:  phylogenetic analysis of bacterial peptides | Diagnosis:  SCID fourth edition  Symptoms:  Hamilton Depression Rating Scale | **Phylogenetic analysis of differential bacterial peptides on the basis of the lowest common ancestor approach**  *Phylum level*  ↑Actinobacteria, Firmicutes derived proteins in MDD  ↓ Bacteroidetes, Proteobacteria derived proteins in MDD  *Family level*  ↑ *Actinomycetaceae*, *Bifidobacteriaceae*, *Clostridiaceae*, *Erysipelotrichaceae, Lachnospiraceae*, *Nocardiaceae*, *Porphyromonadaceae, Ruminococcaceae, Streptomycetaceae* derived proteins in MDD   ↓ *Chitinophagaceae*, *Enterobacteriaceae*, *Mariniabiliaceae*, *Oscillospiraceae*, *Prevotellaceae*, *Sutterellaceae* derived proteins in MDD  ↔ *Rikenellaceae* in MDD (unclear direction of effect based on figure) | | | **Alpha and beta diversity**  Did not analyse  **Other**  Hierarchical cluster analysis  989 differential bacteria peptides in MDD compared to controls  (534 upregulated and 455 downregulated) |
| **11) Chen, Xue et al (2021)**  Case-control, n=108  (MDD, n=62; Controls, n=46 [subset analysed with metagenomics (MDD, n=20; Controls, n=21)]  DOI: 10.1016/j.jad.2020.12.143 | Bacteria:  16s rRNA gene sequencing- Illumina MiSeq platform  Shotgun metagenomic sequencing in a subset | Diagnosis:  Psychiatrists-based using the DSM-V and HAMD-17 score ≥ 18  Symptoms:  Hamilton Depression Rating Scale, Hamilton Anxiety Scale and the Positive and Negative Syndrome Scale | **Linear discriminant analysis and LEfSe of 16S sequencing data**  *Phylum level*  ↑ *Bacteroidetes, proteobaeteria* and *Fusobacteria* in MDD  ↑ *Firmicutes* and *Actinobacteria* in Controls  *Family level*  ↑ *Ruminococcaceae, Lachnospiraceae*, and *Coriobacteriales_unclassified* in Controls  ↑ *Enterobacteriaceae, Tannerellaceae, Burkholderiaceae, Campylobacteraceae, Corynebacteriaceae* and *Clostridia_unclassified* in MDD  *-Genus level (the 20 most significant ones)*  ↑ *Escherichia-Shigella, Parabacteroides,* and *Lachnoclostridium* in MDD  ↑ *Faecalibacterium, Agathobacter, Ruminococcus2, Subdoligranulum, Bifidobacterium, Blautia, Roseburia, Ruminococcus1, Coriobacteriales_unclassified, Ruminococcaceae_UCG-013*, *Dorea*, *Eubacterium_ventriosum_group, Anaerostipes, Butyricicoccus, Lachnospiraceae NK4A136group, Coprococcus3, Fusicatenibacter* in Controls  **Random Forest Classification Models of MDD**  ↑ OTUs from the genus Lachnoclostridium, Escherichia-Shigella, Prevotellaceae_unclassified, Klebsiella, Alistipes in MDD  ↑ OTUs from the genus Faecalibacterium, Butyricicoccus, Agathobacter, Ruminococcaceae_UCG-013, Dorea, Coprococcus_3, Eubacterium ventriosum group, Eubacterium_hallii_group, Subdoligranulum, Tyzzerella, Family_XIII_AD3011_group in Controls  **Metagenome-wide association analysis of the metagenomics**  *Phylum level*  ↑ *Bacteroidetes, Verrucomicrobia* and *Fusobacteria* in MDD  ↑ *Firmicutes* in Controls  *Family level*  ↑ *Ruminococcaceae, Lachnospiraceae*, *Eubacteriaceae* and *Lactobacillaceae* in Controls  ↑ *Rikenellaceae, Porphyromonadaceae, Oscillospiraceae* and *Corynebacteriaceae* in MDD  *-Genus level (the main ones mentioned here)*  ↑ *Barnesiella, Parabacteroides, Alistipes, Flavonifractor, Anaerotruncus, Ruminococcaceae, Campylobacter, Adlercreutzia, Paraprevotella, Clostridiales, Oscillibacter, Bilophila, Akkermansia* and *Gammaretrovirus* in MDD  ↑ *Eubacterium, Dorea, Roseburia, Faecalibacterium, Lactobacillus, Megamonas, Megasphaera and Haemophilus* in Controls  *-Species level (the main ones mentioned here)*  ↑ *Alistipes_onderdonkii, Barnesiella_intestinihominis, Bacteroides_caccae, Oscillibacter_unclassified, Bilophila_unclassified,* and *Alistipes_finegoldii* in MDD  ↑ *Faecalibacterium_prausnitzii, Eubacterium_rectale, Roseburia_inulinivorans*, and *Roseburia_hominis* in Controls | | | **Alpha and beta diversity**  No significant differences in ACE, Chao, Shannon and Simpson indexes bet groups  Significant differences in microbial abundance bet groups ( PCoA)  **Other**  HAMD scores positively correlated with Anaerotruncus, Parabacteroides, and Anaeroglobus and negatively correlated with Fusicatenibacter (for correlation with other clinical parameters refer to the paper)  For functional genes differentially enriched (KEGG database) bet groups refer to the paper |
| **12)**  **Chen, Zheng et al. (2018)**  Case-control study, N=88  (Sex-specific differences in gut microbiota in first-episode drug naive MDD [Females, n=24; Males, n=20] and Matched Controls [Females, n=24; Males, n=20])  DOI: 10.2147/NDT.S159322 | Bacteria:  16S rRNA gene 454 sequencing | Diagnosis:  Psychiatrists-based using Hamilton Depression Rating Scale (17-item version) | Males | | Females | **Alpha and beta diversity**  No significant difference in phylogenetic diversity  Richness and richness/evenness appeared to have been assessed but not reported (observed species, Shannon, Simpson)  MDD clustered separately from controls on both PCoA (distance measure unclear) and partial least squares-discriminant analysis for both males and females (sex-specific analysis) |
|  |  |  | **Random forest algorithm and LEfSe used to identify the differential OTUs**  *Phylum level*  ↓ Bacteroidetes in MDD  *Class level*  ↑ Bacteroidia in MDD  ↓ Clostridia in MDD  *Family level*  ↑ *Bacteroidaceae* in MDD  ↓ *Coriobacteriaceae* in MDD  *Genus level*  ↑ *Atopobium, Bacteroides, Erysipelotrichaceae incertae sedis*, *Veillonella* in MDD ↓ *Anaerovorax*, *Clostridiaceae 1*, *Gordonibacter*, and *Pyramidobacter* in MDD | | **Random forest algorithm and LEfSe used to identify the differential OTUs**  *Phylum level*  ↑ Actinobacteria in MDD  *Order level*  ↑ Bifidobacteriales, Coriobacteriales, Pasteurellales  *Family level*  ↑ *Bifidobacteriaceae, Coriobacteriaceae, Eubacteriaceae, Lachnospiraceae*, *Pasteurellaceae* in MDD  *Genus level*  ↑ *Actinomyces, Anaerostipes, Asaccharobacter, Atopobium, Bifidobacterium, Blautia, Desulfovibrio, Eggerthella, Eubacterium, Faecalibacterium, Gordonibacter, Olsenella, Roseburia* in MDD  ↓ *Howardella, Pyramidobacter*, *Sutterella* in MDD |  |
|  |  |  | **Correlations**  Positive correlations between depression symptoms (HAM-D) and Actinobacteria, Coriobacteriales, *Coriobacteriaceae*, *Collinsella*  Negative correlations between depression symptoms (HAM-D) and Firmicutes, Clostridia, Negativicutes, Clostridiales, Selemonadales, *Lachnospiraceae, Veillonellaceae, Veillonella* | | **Correlations**  Negative correlations between depression symptoms (HAM-D) and Bacilli, Lactobacillales, *Streptococcaceae,* *Clostridium XIVa, Erysipelotrichaceae incertae sedis*, *Streptococcus* |  |
| **13) Chen et al. (2019)** Case-control, n=60  (GAD, n=36; Controls, n=24)  DOI: 10.1016/j.jad.2019.09.033 | Bacteria:  16S rRNA gene sequencing- Illumina MiSeq platform | Diagnosis:  MINI to screen for pre-existing psychiatric disorders  Psychiatrist reported diagnosis (DSM-5)  Symptoms:  Hamilton Anxiety Rating Scale  Hamilton Depression Rating Scale  Self-rating Anxiety Scale  Self-rating Depression Scale | **Taxonomic differences analysed using LEfSe approach**  *Phylum level*  ↓ Firmicutes, Tenericutes in GAD  *Class level*  ↓ Mollicutes in GAD  *Order level*  ↑ Betaproteobacteriales, Enterobacteriales in GAD  *Family level*  ↑ *Bacteroidaceae,* *Burkholderiaceae*, *Enterobacteriaceae* in GAD  ↓ *Muribaculaceae* and *Prevotellaceae* in GAD  *Genus level*  ↑ *Bacteroides, Escherichia/Shigella*, *Hungatella* in GAD  ↓ *Acinetobacter,* *Agathobacter, Buchnera*, *Dialister, Megamonas*, *Subdoligranulum* in GAD  *Species level*  ↑ *Tyzzerella 3* in GAD  ↓ *Clostridium innocuum*, *Coprococcus 1*, *Coprococcus 3*, *Eubacterium coprostanoligenes*, *Eubacterium ruminantium*, *Eubacterium xylanophilum, Lachnospiraceae NK4A136*, *Mollicutes RF39*, *Prevotella 9*, *Ruminococcaceae NK4A214, Ruminococcaceae UCG-014* in GAD | | | **Alpha and beta diversity**  ↓ Richness on two indices (ACE, observed OTUs) but trend only in another (Chao1, p = .094) in GAD  No significant difference in richness/evenness (Shannon, Simpson)  GAD clustered separately from controls on PCoA (weighted and unweighted UniFrac) |
|  |  |  | **Taxonomic differences analysed using Metastats**  *Phylum level*  ↓ Firmicutes, Tenericutes in GAD  *Family level*  ↑ *Bacteroidaceae*, *Burkholderiaceae,* *Enterobacteriaceae* in GAD  ↓ *Muribaculaceae*, *Prevotellaceae,* *Succinivibrionaceae* in GAD  *Genus level*  ↑ *Bacteroides, Escherichia/Shigella, Raoultella* in GAD  ↓ *Acinetobacter, Agathobacter, Buchnera, Dialister, Holdemanella, Megamonas, Mitsuokella, Subdoligranulum, Succinivibrio* in GAD  *Species level*  ↑ *Clostridium innocuum* in GAD  ↓ *Coprococcus 3, Eubacterium coprostanoligenes, Eubacterium ruminantium, Lachnospiraceae NK4A136, Mollicutes RF39, Muribaculaceae norank, Prevotella 9, Prevotellaceae NK3B31, Prevotellaceae UCG-001, Ruminococcaceae NK4A214, Ruminococcaceae UCG-014, Tyzzerella 3* in GAD | | |  |
|  |  |  | **Correlation analysis**  Consistent positive correlations between: -  *- Escherichia-Shigella* and all anxiety and depression scale scores (HAM-A, somatic anxiety, mental anxiety, SAS, HAM-D, SDS)  *- Bacteroides* and some anxiety (somatic anxiety, SAS) and both depression scales (HAM-D, SDS)  - *Veillonella* and majority of anxiety scales (HAM-A, mental anxiety)  Consistent negative correlations between: -  *- Mitsuokella*, *Succinivibrio*, *Eubacterium coprostanoligenes*, *Mollicutes RF39*, *Prevotella 9*, *Prevotellaceae UCG-001*, *Ruminococcaceae NK4A214, Ruminococcaceae UCG-014* and all anxiety and depression scale scores (HAM-A, somatic anxiety, mental anxiety, SAS, HAM-D, SDS)  *- Subdoligranulum* and all but one anxiety/depression scale (HAM-A, somatic anxiety, mental anxiety, HAM-D, SDS)  - *Lachnospiraceae NK4A136* and one anxiety (HAM-A) plus both depression scales (SDS, HAM-D)  A number of other genera/species were negatively associated with some questionnaires but not others: -  *- Agathobacter* (HAM-A, somatic anxiety)  *- Dialister, Prevotellaceae NK3B31* (somatic anxiety, SAS, SDS)  *- Megamonas, Christensenellaceae R-7* (somatic anxiety, HAM-D)  *- Coprococcus 3, Muribaculaceae_no rank, Ruminococcaceae UCG-002* (SAS)  *- Eubacterium ruminantium* (SDS)  *- Eubacterium ventriosum* (HAM-A, SAS, SDS)  - *Ruminococcus 2* (SAS, SDS) | | |  |
| **14) Chung et al. (2019)** Case-control, n=73 (MDD, n=36 and Controls, n=37)  DOI: 10.1016/j.jpsychires.2019.01.016 | Bacteria:  16S rRNA gene sequencing- Illumina MiSeq/MiniSeq platforms | Diagnosis:  Psychiatrist reported diagnosis (DSM-5)  SADS-LA  Symptoms:  Beck Depression Inventory  Beck Anxiety Inventory | **Applied analysis of composition of microbiomes (ANCOM), adjusting for fat intake and sequencing platform**  *Phylum level*  ↑ Actinobacteria, Firmicutes in MDD  ↓ Bacteroidetes, Proteobacteria in MDD  *Family level*  ↑ *Bifidobacteriaceae, Lachnospiraceae,* *Peptostreptococcaceae*, *Porphyromonadaceae*, *Streptococcaceae* in MDD  ↓ *Alcaligenaceae* and *Prevotellaceae* in MDD  *Genus level*  ↑ *Adlercreutzia*, *Bifidobacterium, Blautia, Clostridium XI*, *Eggerthella*, *Holdemania*, *Parabacteroides*, *Ruminococcus*, *Streptococcus* in MDD  ↓ *Megamonas*, *Prevotella, Sutterella* in MDD | | | **Alpha and beta diversity**  No significant differences in:  - Richness (Chao1, observed OTUs)  - Richness/evenness (Shannon)  - Phylogenetic diversity (Whole Tree)  MDD clustered separately from controls on PCoA/ PERMANOVA (weighted and unweighted UniFrac) |
|  |  |  | **Associations in the whole sample**  Positive correlations between:  *Family level*  - *Peptostreptococcaceae* and *Porphyromonadaceae* with depression symptoms (BDI)  - *Porphyromonadaceae* with anxiety symptoms (BAI)  *Genus level*  - *Blautia*, *Clostridium XI, Eggerthella, Parabacteroides*, and *Ruminococcus* with depression symptoms (BDI)  - *Eggerthella* and *Parabacteroides* with anxiety symptoms (BAI)  - *Holdemania* with anxiety symptoms (BAI) in MDD only  Negative correlations between:  - *Family level: Alcaligenaceae* and *Prevotellaceae* with depression symptoms (BDI)  - *Genus level: Prevotella* and *Sutterella* with depression symptoms (BDI) | | |  |
| **15) Dong et al. (2021)**  Case-control, n=54 (MDD, n=23; GAD, n=21 and Controls, n=10)  DOI: 10.3389/fpsyt.2021.651536 | Bacteria:  16S rRNA gene sequencing- Illumina | Diagnosis:  Psychiatrics diagnosis with DSM-V  Symptoms:  Hamilton Depression Rating Scale (HAMD-24) and the Hamilton Anxiety Scale (HAMA) | **Levels of OTUs altered among the three groups**  ↓ Otu24167, Otu19140, and Otu19751in MDD vs Controls  ↑ Otu2563 in MDD vs GAD or Controls  ↑ Otu2581 and Otu10585 in GAD vs MDD  No significant difference in the abundance of OTUs between GAD and Controls  - *Genus level:*  ↓ Sutterella and Fusicatenibacter in MDD vs Controls  ↓ Fusicatenibacter and Christensenellaceae_R7_group in GAD vs Controls  ↑ Sutterella in GAD vs MDD  ↓ Faecalibacterium in GAD vs MDD | | | **Alpha and beta diversity**  No significant difference in richness and diversity bet MDD and Controls  Significant differences in Ace, Chao, Simpson and Shannon bet GAD and Controls  Beta diversity analysis showed similar species diversity among the three groups  **Other**  Christensenellaceae_R7_group negatively correlated with the HAMD factor score (Limited to Hopelessness) and total score  (refer to the paper for other results |
| **16) Dong et al. (2022)**  Case-control, n=93 (MDD, n=63; Controls, n=30)  8-week intervention with antidepressant treatment  DOI: 10.3389/fnins.2022.813075 | Bacteria:  16S rRNA gene sequencing- Illumina | Diagnosis:  Structured Clinical Interview for DSM-IV (SCID) by two psychiatrists  Symptoms:  HAMD-24 before and after treatment | **MDD vs Controls**  *Phylum level*  ↑ Actinobacteria in MDD  *Family level*  ↑ Bifidobacteriaceae in MDD and ↓ Lactobacillaceae in MDD  *Genus level:*  ↑ *Bifidobacterium, Blautia*, and *Agathobacter* in MDD  **Responder and non-responder groups (classified based on HAMD values) at baseline**  *Phylum level*  ↓ Actinobacteria in responders  *Family level*  ↓ Christensenellaceae and Eggerthellaceae in responders  *Genus level:*  ↓ *Adlercreutzia* and *Christensenellaceae R7 group* in responders | | | **Alpha and beta diversity**  No statistical significant differences in Ace, Chao1, Simpson, and Shannon indices  No statistical significant differences in β-diversity estimates (PCoA and ANOSIM based on Bray–Curtis distances)  **Other**  For results on metabolites refer to the paper |
| **17) Fontana et al. (2020)**  Case-control, n=54 (MDD, n=34; Controls, n=20), DOI: 10.3390/biomedicines8090311 | Bacteria:  16S rRNA gene sequencing- Illumina MiSeq | Diagnosis:  DSM IV-TR | **Comparison of Gut Microbiota Composition between MDD Patients with and without treatment resistant and Controls-penalized logistic regression analysis**  ↑ *Elusimicrobia, Flavobacteriaceae, Fenollaria* and *Robinsoniella sp. MCWD5* in responder MDD  ↑ *Nitrospirae* and *Peptostreptococcaceae* in Controls  ↑ *Proteobacteria* in Controls best discriminating bet responders and Controls  ↑ Flavobacteriaceae, Hungatella, Yersinia, Citrobacter, Fenollaria and Fenollaria timonensis in treatment-resistant MDD & ↑ Candidatus Saccharibacteria and Massilioclostridium coli in Controls were the bacterial patterns distinguishing these groups | | |  |
| **18) Gao et al. (2022)**  Case-control, n=175  Frontline healthcare workers (FHWs) isolated for 2mos due to COVID-19, n=71; Second-line healthcare workers (SHWs) working in hospitals, n=104  Longitudinal associations from baseline (day 1 of isolation) to follow up (day 180 last day of isolation) on 52 FHWs who completed 180 days of follow-up  DOI: 10.1016/j.jad.2022.02.024 | Bacteria:  16S rRNA gene sequencing-full-length | Symptoms:  9-item Patient Health Questionnaire, the 7-item Generalised Anxiety Disorder Scale | **Longitudinal associations with species selected by cross-validated random forest models**  Most species were from the genus [Eubacterium] eligens group, Bacteroides, Faecalibacterium, Lachnospiraceae NK4A136 group, and Streptococcus in Cluster 6 (genera with continuing decline). Other common species were from Bifidobacterium and Sutterella in Cluster 4 (genera with lowest relative abundance at Day 45), and the Lachnospiraceae ND3007 group in Cluster 3 (genera that increased from Day 45 to Day 180)  Species associated with the 7-item Generalised Anxiety Disorder Scale  *Lactobacillus ruminis ATCC 25644, Bifidobacterium adolescentis ATCC 15703, Heamophilus parainfluenzae T3T1, Heamophilus unclassified, Sutterella wadsworthensis, Sutterella uncultured bacterium, [Eubacterium] xylanophilum group_uncultured bacterium, Klebsiella pneumoniae, [Eubacterium] eligens ATCC 27750, [Eubacterium] eligens group unclassified, [Eubacterium] eligens group_uncultured bacterium, Bacteroides coprophilus, Bacteroides finegoldii, Bacteroides plebeius, Bacteroides uniformis, Feacalibacterium gut metagenome, Feacalibacterium human gut metagenome, Feacalibacterium unclassified, Lachnospiraceae NK4A136 group uncultured bacterium, Lachnospiraceae uncultured bacterium, Streptococcus anginosus C1051, Streptococcus salivarius, Streptococcus sp. FDAARGOS_192*  Species associated with the 9-item Patient Health Questionnaire  *[Eubacterium] hallii group unclassified, Lactobacillus ruminis ATCC 25644, Alistipes finegoldii, Bifidobacterium adolescentis, Bifidobacterium adolescentis ATCC 15703, Bifidobacterium unclassified, Heamophilus parainfluenzae T3T, Sutterella wadsworthensis, Sutterella uncultured bacterium, Citrobacter unclassified, Megamonas_uncultured bacterium, [Eubacterium] eligens ATCC 27750, Bacteroides coprophilus, Bacteroides finegoldii, Bacteroides massiliensis, Bacteroides plebeius, Bacteroides uniformis, Bacteroides unclassified, Feacalibacterium prausnitzii, Feacalibacterium gut metagenome, Feacalibacterium unclassified, Lachnospiraceae bacterium GAM79, Lachnospiraceae NK4A136 group uncultured bacterium, Prevotella_9 unclassified, Streptococcus anginosus C1051, Streptococcus salivarius* | | | **Alpha and beta diversity**  Significant differences in Chao1 and number of observed amplicon sequence variates (ASVs) bet FHWs and SHWs  Significant differences in beta-diversity (PCoA based on the Bray-Curtis matrix, Mann-Whitney U test) bet FHWs and SHWs |
| **19) Gao et al. (2023)**  Case-control, n=103  (MDD, n=62; Matched controls, n=41)  Intervention:  antidepressants for 8 weeks [(treatment-resistant (TR), n=25; or responders (R), n=37] DOI: 10.1016/j.jad.2023.02.143 | Bacteria:  16S rRNA gene sequencing- Illumina MiSeq | Diagnosis:  DSM-V  Symptoms:  7-item Hamilton Depression Rating Scale | **LEfSe**  Genus level  ↑ *Bacteroides, Roseburia, Faecalibacterium, Dialister, Megasphaera, Ruminococcus, Phascolarctobacterium, Parabacteroides, Oscillospira, Sutterella, Lachnospira, Haemophilus* in Controls  ↑ *Blautia, Bifidobacterium, Coprococcus, Collinsella,* and *Dorea* in R  ↑ *Megamonas* and *ph 2* in TR  *Blautia, Bifidobacterium, Coprococcus* were positively correlated with the effect of SSRI antidepressants | | | **Alpha and beta diversity**  Significant differences in the Goods coverage, Shannon, Simpson and Pielou e bet the three groups (TR, R and Controls)  Significant difference in beta bet R and Controls (PCoA based on Jaccard dissimilarity) |
| **20) Guo et al. (2022)**  Case-control, n=125  [GAD, n=44; 16 functional gastroenteropathy (FGID); Controls (CG), n=30; comorbid GAD and FGID (FAD), n=35]  DOI: 10.3389/fpsyt.2022.946808 | Bacteria:  16S rRNA gene sequencing- Illumina | Diagnosis:  ICD-11  Symptoms:  Hamilton Anxiety Scale (HAMA-14) | **LEfSe**  ↓ *Ruminococcaceae, Faecelibacterium,* and *Agathobacter* in the FAD vs CG group  ↑ Fusobacterium in the FAD vs GAD  ↑ Fusobacterium and Bacteroid in the FAD vs FGID group  ↓ Pasteurella, Hemophilus, Parainfluenzae, and Hemophilus in the FAD vs FGID  ↑ Pasteurella, Hemophilus, Parainfluenzae, and Hemophilus FGID vs GAD  ↑ Enterobacterium, Pasteurella, Haemophilus, Parainfluenzae, and Haemophilus in FGID vs CG  ↓ Aathobacter in FGID vs CG  ↓ Faecalibacterium in the GAD vs CG  ↑ Megamonas in the GAD vs CG  **Correlations**  HAMA significantly positively correlated with *Fusobacterium, Veillonella*, *Megamonas,* Bacteroides and Enterobacteriaceae. HAMA negatively correlated with Faecalibacterium and Ruminococcaceae | | | **Alpha and beta diversity**  No significant difference in species richness among groups ( chao1 index and PD whole_tree)  Differences in the composition of intestinal flora between the four groups (no statistical test) |
| **21) Han et al. (2023)**  Case-control, n=81  (MDD, n=51; Controls, n=30)  DOI: 10.3389/fgene.2022.976814 | Bacteria:  16S rRNA gene sequencing- Illumina | Diagnosis:  Structured Clinical Interview for DSM-IV by psychiatrists | **LEfSe**  Order level  ↑ Actinobacteria and Pseudomonadales in MDD  ↓ Rhizobiales, Mollicutes_RF39, Tenericutes, and Mollicutes in MDD group  Genus level  ↑ *Actinobacteria, Ruminococcus gravus group, Eggerthella, Sellimonas, Moraxellaceae, Pseudomonadales* in MDD  ↓ *Lactobacillus, Rombutsia, Adlercreutzia, Mollicutes, Terenicutes, Mollicutes_RF39* in MDD | | | **Alpha and beta diversity**  ACE and Chao were significantly higher in Controls. No difference in Shannon and Simpson  PCoA showed no significant clustering between the cohorts |
| **22) Heym et al. (2019)**  Cross-sectional associations, n=40  DOI: 10.1007/s00213-019-05230-2 | Bacteria:  16S rRNA-targeted oligonucleotide probes for Bifidobacterium spp. (Bif164) and Lactobacillus spp. (Lab158) | Symptoms:  Beck Depression Inventory-II | Linear regression revealed that *Lactobacillus* was associated with positive self-judgement, but not cognitive depression in model also including age, sex, cognitive depression, over-identification, affective empathy  No associations between psychometric properties and *Bifidobacterium* spp. | | | **Alpha and beta diversity**  Did not analyse  **Other**  Associations between inflammatory markers and *Lactobacillus* and *Bifidobacterium spp.* Also examined positive self-judgment and empathy |
| **23) Hope et al. (2023)**  Case-control, n=20  (With depressive symptoms, n=11; Without, n=9)  DOI: 10.1177/10998004221124273 | Bacteria:  16S rRNA gene sequencing | Symptoms:  Patient-Reported Outcomes Measurement Information System (PROMIS) Short Form–Depression | **LEfSe**  Phylum level  ↑ Actinobacteria in participants with depressive symptoms  ↑ Bacteroidetes in participants without depressive symptoms  **LEfSe to distinguish level of depressive symptoms (No vs. Mild vs. Moderate/severe)**  ↑ Lactobacillales (order), Bacteroides (genus), Bacteroidaceae (family) and Actinobacteria (phylum) in participants moderate/severe depressive symptoms  ↑ Prevotellaceae (family) in participants with mild depressive symptoms  ↑ Bacteroidetes (phylum), Bacteroidia (class), Bacteroidales (order), Rikenellaceae (family), and genera Alistipes and Holdemania in participants without depressive symptoms | | | **Alpha and beta diversity**  α-diversity was marginally associated with a higher level of depressive symptoms (Chao1, p = 0.09] and Shannon, p = 0.08)  Weighted UniFrac distance showed dissimilarities between groups |
| **24) Huang et al. (2018)** Case-control study, n=54 (MDD, n=27 and Controls, n=27)  DOI: 10.2147/NDT.S188340 | Bacteria:  16S rRNA gene sequencing- Illumina HiSeq2500 | Diagnosis:  ICD-10 | **Metagenomes predicted using PICRUST**  *Phylum level*  ↓ Firmicutes in MDD  *Family level*  ↓ *Clostridiaceae, Lachnospiraceae*, *Ruminococcaceae* in MDD  *Genus level*  ↑ *Bulleidia*, *Gemella,* *Oxalobacter*, *Parvimonas*, *Peptostreptococcus*, *Pseudomonas* in MDD  ↓ *Blautia,* *Coprococcus*, *Dorea, Faecalibacterium* in MDD | | | **Alpha and beta diversity**  ↓ Richness (Chao1, ACE) in MDD  ↓ Richness/ evenness (Shannon) in MDD  ↓ Phylogenetic diversity (Faith’s) in MDD  MDD clustered separately from controls on PCoA (weighted UniFrac), however less obvious clustering using unweighted UniFrac |
| **25) Jackson et al. (2018)**  Cross-sectional associations, N = 2737 (Depression and anxiety), DOI: 10.1038/s41467-018-05184-7 | Bacteria:  16S rRNA gene sequencing- Illumina MiSeq platform | Diagnosis: Self-reported diagnosis of depression or anxiety by a doctor or health professional | **Beta coefficient matrix using cosine similarity**  *Class level*  ↓ Mollicutes in anxiety  *Family level*  ↑ *Lachnospiraceae, Streptococcaceae* in depression  ↑ *Micrococcaceae*, *Streptococcaceae* in anxiety  ↓ *Bifidobacteriaceae*, *Odoribacteraceae,* *Peptostreptococcaceae*, *S24.7*, *Turicibacteraceae* (*Erysipelotrichaceae*) in depression  ↓ *Peptococcaceae, S24.7* in anxiety | | | **Alpha and beta diversity**  No association between richness/evenness (Shannon) or phylogenetic diversity with anxiety or depression  Generated cosine distances for top 6 principle coordinates revealed associations with β-diversity in both depression and anxiety |
| **26) Jiang et al. (2015)**  Case-control study, n=76 (Active-MDD, n=29; Responding-MDD, n=1; Controls, n=30)  DOI: 10.1016/j.bbi.2015.03.016 | Bacteria:  16S rRNA gene 454 sequencing- Roche | Diagnosis:  MINI to detect pre-existing psychiatric disorders  MDD verified using SCID, fourth edition  Symptoms:  Hamilton's Depression Scale  Montgomery–Åsberg Depression Rating Scale | **Taxonomic differences analysed using LEfSe approach between MDD and controls**  *Order level*  ↑ Enterobacterales in MDD  ↓ Pasteurellales in MDD  *Family level*  ↑ *Enterobacteriaceae*, *Porphyromonadaceae*, *Rikenellaceae* in MDD  ↓ *Pasteurellaceae*, *Peptostreptococcaceae, Ruminococcaceae* in MDD  *Genus level*  ↑ *Alistipes*, *Butyricimonas, Clostridium XIVb*, *Erysipelotrichaceae unclassified, Flavonifractor, Parabacteroides*, *Porphyromonadaceae* *unclassified* in MDD  ↓ *Dialister, Escherichia/Shigella, Faecalibacterium, Haemophilus, Ruminococcus* in MDD | | | **Alpha and beta diversity**  ↑ Richness/evenness in active-MDD group on one index (Shannon) but no difference using another (Simpson)  No difference in richness (ACE, Chao1) or evenness (Shannon index of evenness)  No significant difference between MDD and controls on PCoA (unweighted UniFrac)  **Other**  Negative correlation between *Faecalibacterium* and depressive symptoms (HAM-D)  Also examined associations with remitted MDD |
|  |  |  | **Differences between MDD vs Controls using Metastats**  *Phylum level*  ↑ Bacteroidetes, Fusobacteria, Proteobacteria in MDD  ↓ Actinobacteria, Firmicutes in MDD  *Family level*  ↑ *Acidaminococcaceae*, *Enterobacteriaceae*, *Fusobacteriaceae*, *Porphyromonadaceae*, *Rikenellaceae* in MDD  ↓ *Bacteroidaceae*, *Erysipelotrichaceae*, *Lachnospiraceae*, *Prevotellaceae*, *Ruminococcaceae* (not in diagram but in text), *Veillonellaceae* in MDD  *Genus level*  ↑ *Alistipes, Blautia, Clostridium XIX, Lachnospiraceae incertae sedis, Megamonas, Oscillibacter, Parabacteroides, Parasutterella, Phascolarctobacterium, Roseburia* in MDD  ↓ *Bacteroides, Dialister, Faecalibacterium, Prevotella*, *Ruminococcus* in MDD | | |  |
| **27) Jiang et al. (2018)** Case-control study, n=76  (GAD, n=40; Controls, n=36)  DOI: 10.1016/j.jpsychires.2018.07.007 | Bacteria:  16S rRNA gene sequencing- Illumina MiSeq platform | Diagnosis:  MINI for systematic psychiatric screening  GAD verified using SCID, fourth edition  Symptoms:  Hamilton Anxiety Rating Scale | **Differential abundance examined using PERMANOVA of unweighted UniFrac PCoA** **(bold** indicates findings also observed in medication naïve subset)  *Phylum level*  ↑ Bacteroidetes, Fusobacteria in GAD  ↓ Firmicutes in GAD  *Genus level*  ↑ ***Bacteroides, Fusobacterium*** in GAD  ↓ *Butyricicoccus,* ***Faecalibacterium****,* ***Lachnospira,*** *Sutterella* in GAD  Only observed in medication naïve participants:  ↑ *Escherichia/Shigella, Lactobacillus* in GAD  ↓ *Roseburia, Subdoligranulum* in GAD  *Species level*  ↑ ***Ruminococcus gnavus*** in GAD  ↓ ***Eubacterium rectale*** in GAD A number of results were inconsistent in prose and in figures. Given consistency across figures in the medication naïve and group on medication, results reported above reflect figure data.  **Differential abundance between GAD and controls analysed using LEfSe (bold** indicates findings also observed in medication naïve subset)  *Phylum level*  ↑ **Bacteroidetes, Fusobacteria** in GAD  ↓ **Firmicutes** in GAD  *Class level*  ↑ **Bacteroidia** in GAD  ↓ **Clostridia**, Betaproteobacteria, Deltaproteobacteria in GAD  *Order level*  ↑ **Bacteroidales, Fusobacteriales** in GAD  ↓ **Clostridiales**, **Coriobacteriales,** Burkholderiales, Desulfovibrionales in GAD Only in medication naïve participants: ↑ Enterobacteriales in GAD  *Family level*  ↑ ***Bacteroidaceae, Fusobacteriaceae*** in GAD  ↓ ***Christensenellaceae***, ***Coriobacteriaceae*, *Lachnospiraceae***, ***Peptococcaceae***, ***Ruminococcaceae,*** *Alcaligenaceae,* *Desulfovibrionaceae* in GAD  Only in medication naïve participants:  ↑ *Enterobacteriaceae*, *Lactobacillaceae* in GAD  ↓ *Clostridiales* *vadin BB60, Family* *XIII*, *Oxalobacteraceae*, *Prevotellaceae* in GAD  *Genus level*  ↑ ***Bacteroides,*** *Eisenbergiella,* ***Erysipelatoclostridium****,* ***Flavonifractor****,* ***Fusobacterium*** in GAD  ↓ *Bilophila,* ***Butyricicoccus,*** *Clostridium sensu stricto, Dialister,* ***Faecalibacterium***, ***Lachnospira,*** *Pseudobutyrivibrio,* ***Roseburia****,* ***Subdoligranulum***, *Sutterella* in GAD  Only in medication naïve participants:  ↑ *Escherichia/Shigella, Lactobacillus, Parabacteroides* in GAD  ↓ *Anaerotruncus, Barnesiella*, *Butyricimonas, Collinsella, Fusicatenibacter*, *Moryella, Oxalobacter, Parasutterella, Peptococcus* in GAD  *Species level*  ↑ ***Clostridium innocuum,*** *Lachnospiraceae unclassified,* ***Ruminococcus gnavus****,* ***Tyzzerella 4*** in GAD  ↓ ***Christensenellaceae R7****, Clostridiaceae 1,* ***Coprococcus 1****,* ***Coprococcus 2****,* ***Eubacterium eligens****,* ***Eubacterium rectale****,* ***Family XIII UCG-001***, ***Lachnospiraceae FCS020***, ***Lachnospiraceae NC2004****,* ***Lachnospiraceae ND3007****,* ***Lachnospiraceae UCG-001****,* ***Lachnospiraceae UCG-004****,* ***Ruminococcaceae NK4A214****,* ***Ruminococcaceae UCG-003****,* ***Ruminococcaceae UCG-005****,* ***Ruminococcaceae UCG-010****,* ***Ruminococcaceae unclassified****,* ***Ruminococcus gauvreauii group*** in GAD  Only in medication naïve participants:  ↑ *Ruminococcaceae UCG-014, Ruminococcus torques* in GAD  ↓ *Eubacterium coprostanoligenes, Eubacterium ruminantium, Eubacterium ventriosum, Eubacterium xylanophilum,* *Family XIII AD3011, Lachnospiraceae NK4A136*, *Lachnospiraceae UCG-010, Lachnospiraceae unclassified, Ruminococcaceae UCG-002, Ruminococcus 1* in GAD | | | **Alpha and beta diversity**  ↓ Richness in GAD on one index (observed OTUs) but trend only in another (Chao1, *p* = .062)    No significant differences in richness/evenness (Shannon, Simpson)  **Treatment naïve subset:**  ↓ Richness (ACE, number of observed OTUs, Chao1)    ↔ Richness/evenness (Simpson reported as decreased in text but increased in figure)  GAD clustered separately from controls on PCoA/ PERMANOVA (unweighted UniFrac), with a trend observed for treatment-naïve subset (*p* = .06)  Sex had no significant effect on the α-diversity, β-diversity or relative abundance of associated GAD taxa |
| **28) Kelly et al. (2016)** Case-control study, n=67  (MDD, n=34; controls, n=33)  DOI: 10.1016/j.jpsychires.2016.07.019 | Bacteria:  16S rRNA gene sequencing- Illumina MiSeq platform | Diagnosis:  Controls screened using MINI (DSM-IV)  DSM-IV used to confirm MDD diagnosis  Symptoms:  Beck Depression Inventory  Hamilton Depression Rating Scale (17-item version)  Beck Anxiety Inventory | *Family level*  ↑ *Thermoanaerobacteriaceae* in MDD  ↓ *Prevotellaceae* in MDD  *Genus level*  ↑ *Anaerofilum, Eggerthella, Gelria, Holdemania, Paraprevotella*, *Turicibacter* in MDD  ↓ *Dialister, Prevotella* in MDD | | | **Alpha and beta diversity**  ↓ Richness (Chao1, total observed species) in MDD  ↓ Phylogenetic diversity (Faith’s PD) in MDD  No significant difference in richness/evenness (Shannon)  MDD clustered separately from controls on Adonis PERMANOVA/ PCoA (Bray-Curtis,  weighted and unweighted UniFrac)  Statistics based on random permutations on redundancy analysis separated at genus level participants with MDD from the control group |
| **29) Kim et al (2022)**  Case-control, n=39 (Social exclusion, n=14; Controls, n=25)  DOI: 10.1038/s41398-022-02023-8 | Bacteria:  16S rRNA gene pyrosequencing | Symptoms:  Beck Anxiety Inventory (scores) and Beck Depression Inventory (scores) | **Differential abundance compared using independent t-test or Fisher’s exact tests**  *Phylum level*  ↓ Firmicutes/Bacteroidetes ratio in the exclusion group  *Genus level*  ↓ *Faecalibacterium* in the exclusion group  *Faecalibacterium* was not significantly correlated with Beck Anxiety Inventory or Beck Depression Inventory (scores) | | | **Alpha and beta diversity**  Not analysed |
| **30) Kim, Park et al (2022)**  Cross-sectional associations, n=1238  Case-control  Depressed, n=167;  non-depressed, n=1071  DOI: 10.1097/PSY.0000000000001111 | Bacteria:  16S rRNA gene sequencing- Illumina MiSeq | Symptoms:  Center for Epidemiologic Studies Rating Scale for Depression | **Association with the depressed group based on a MaAsLin analysis adjusted for age, sex, and BMI**  ↑ p_Firmicutes; c_Clostridia; o_Oscillospirales; f_Oscillospiraceae; g_Oscillospira in depressed  ↓ p_Firmicutes; c_Clostridia; o_Oscillospirales; f_Ruminococcaceae; g_Faecalibacterium in depressed  (p_ = phylum; c_ = class; o_ = order; f_ = family; g_ = genus)  Significance was lost after adjusting for intake of total energy, carbohydrate, protein, and fat | | | **Alpha and beta diversity**  Depressive symptoms inversely associated with Shannon  Difference bet depressed and non-depressed in the Bray-Curtis dissimilarity |
| **31) Kleiman et al. (2017)** Cross-sectional associations with symptom measures, n=91  DOI: 10.1371/journal.pone.0170208 | Bacteria:  16S rRNA gene sequencing- Illumina MiSeq desktop sequencer | Symptoms:  Beck Anxiety Inventory  Beck Depression Inventory-II | No associations between psychiatric measures and intestinal microbiota composition | | | **Alpha and beta diversity**  No associations between psychiatric measures and richness/evenness (Shannon)  PCoA (unweighted UniFrac) did not show evidence of clustering according to anxiety or depression symptoms |
| **32) Kovtun et al. (2022)**  Case-control, n=74  (Inpatients MDD, n=36; Controls, n=38)  DOI: 10.3390/biomedicines10092162 | Whole metagenome sequencing | Diagnosis:  Depressive outbreaks of medium or high severity according to ICD-10 and 17-item Hamilton Depression ≥14, Center for Epidemiological Studies Depression ≥ 27 and Generalized Anxiety Disorder <10 | **Significantly different bet MDD and Controls after FDR correction**  *Genus level*  *Faecalibacterium, Roseburia, Escherichia, Lachnospira, Ruthenibacterium, Veillonella, Erysipelatoclostridium, Gardnerella, Actinomyces, Intestinibaculum, Faecalibaculum, Pseudobutyrivibrio, Kocuria, Trueperella, Lancefieldella, Fructilactobacillus*  *Species level*  *Roseburia intestinalis, Lactobacillus sanfranciscensis, Veillonella atypica, Ruthenibacterium lactatiformans, Faecalibacterium prausnitzii, Veillonella dispar, Christensenella sp. Marseille-P3954, Pseudobutyrivibrio xylanivorans, Veillonella sp. T1-7, Faecalibaculum rodentium, Intestinibaculum porci, Lancefieldella parvula, Leuconostoc mesenteroides, Gardnerella vaginalis, Roseburia hominis, Lachnospira eligens, Ligilactobacillus ruminis, Phocaeicola salanitronis, Porphyromonas asaccharolytica, Trueperella pyogenes*  *Kocuria varians, Veillonella parvula, Olsenella uli, Escherichia coli, Actinomyces oris, Anaerostipes caccae*  **Significantly different bet MDD and Controls after FDR correction and with > 0.5% abundance**  *Genus level*  ↓ *Faecalibacterium, Lachnospira,* and *Roseburia* in MDD  ↑ *Escherichia* and *Rutenibacterium* in MDD  *Species level*  ↓ *Faecalibacterium prausnitzii, Lachnospira eligens, Roseburia hominis, Roseburia intestinalis, Veillonella atypica, Veillonella dispar, Veillonella parvula,* and *Veillonella sp. T1-7* in MDD  ↑ *Escherichia coli* and *Rutenibacterium* *lactatiformans* in MDD | | | **Alpha and beta diversity**  No significant results for α (Shannon)  Difference at the ‘Species’ level between the two groups  **Other**  For metagenomics signatures refer to the paper |
| **33) Lai et al. (2019)** Case-control study, n=55  (MDD, n=26; Controls, n=29)  DOI: 10.1017/S0033291719003027 | Shotgun metagenomics- Illumina HiSeq2500 | Diagnosis:  MINI for systematic psychiatric screening  MDD verified using SCID-5-CV (clinician version)  Symptoms:  Hamilton Depression Rating Scale (17-item version)  Hamilton Anxiety Rating Scale  Hypomania Checklist (HCL-32) | **Taxonomic differences analysed using LEfSe approach**  *Phylum level*  ↑ Actinobacteria in MDD  ↓ Bacteroidetes in MDD  *Class level*  ↑ Actinobacteria, Coriobacteriia, Negativicutes in MDD  ↓ Bacteroidia, Cytophagia, Flavobacteriia, Sphingobacteria in MDD  *Order level*  ↑ Acidaminococcales, Bifidobacteriales, Coriobacteriales, Eggerthellales, Lactobacillales, Micrococcales, Veillonellales in MDD  ↓ Alteromonadales, Bacteroidales, Cytophagales, Flavobacteriales, Sphingobacteriales in MDD  *Family level*  ↑ *Acidaminococcaceae, Atopobiaceae,* *Bifidobacteriaceae*, *Eggerthellaceae*, *Enterococcaceae*, *Heliobacteriaceae*, *Lactobacillaceae*, *Micrococcaceae*, *Oscillospiraceae*, *Peptococcaceae*, *Veillonellaceae* in MDD  ↓ *Bacteroidaceae*, *Cytophagaceae*, *Flavobacteriaceae*, *Sphingobacteriaceae* in MDD  *Genus level*  ↑ *Acidaminococcus*, *Atopobium*, *Bifidobacterium, Coriobacterium*, *Desulfitobacterium*, *Eggerthella*, *Enterococcus*, *Heliobacterium, Lachnoclostridium*, *Lactobacillus, Megasphaera*, *Streptococcus, Olsenella*, *Oscillibacter*, *Rothia, Slackia*, *Sphaerochaeta* in MDD  ↓ *Bacteroides*, *Sphingobacterium* in MDD  *Species level*  ↑ *Acidaminococcus fermentans, Acidaminococcus intestini, Atopobium parvulum, Bifidobacterium adolescentis, Bifidobacterium bifidum, Bifidobacterium breve, Bifidobacterium dentium, Bifidobacterium longum, Clostridium saccharolyticum, Coriobacterium glomerans, Desulfovibrio vulgaris, Eggerthella lenta, Enterococcus faecium, Eubacterium limosum, Heliobacterium modesticaldum, Lactobacillus crispatus, Megasphaera elsdenii, Olsenella uli, Oscillibacter valericigenes, Rothia mucilaginosa, Slackia heliotrinireducens, Streptococcus parasanguinis, Streptococcus pyogenes, Treponema brennaborense* in MDD  ↓ *Bacteroides helcogenes* in MDD | | | **Alpha and beta diversity**  ↓ Richness/evenness in MDD with one index (Fisher) but trend only in another (Shannon, *p* = .066)  No significant difference in richness/evenness (Fisher, Shannon) between depression subgroups (by medication: SSRI vs SNRI vs drug-free)  MDD clustered separately from controls on PCoA/ PERMANOVA (Bray-Curtis)  No significant difference in β-diversity between medication subgroups in depression  Random forest classification at the genus level and species level achieved area under the curve of .89 and .99, respectively.  Also examined Kyoto Encyclopedia of Genes and Genomes (KEGG) Orthology pathways. |
| **34) Li et al. (2022)**  Case-control, n=62  (Depressed, n=40; Controls, n=22)  DOI: 10.1155/2022/6334868 | Bacteria:  16S rRNA gene sequencing | Diagnosis:  ICD-10 and Hamilton Depression Scale score ≥ 20 | *Species level*  ↑ *Coprococcus catus* in the depressed group  ↓ *Bacteroides barnesiae* in the depressed group | | | **Alpha and beta diversity**  ↓ Shannon, ACE and Chao in depressed  ↑ Simpson in depressed |
| **35) Li, Zhang et al. (2022)**  Case-control, n=291  (MDD, n=120; Control, n=171)  (Sex-specific differences in gut microbiota [Females, n=77; Males, n=43] and Controls [Females, n=71; Males, n=100])  DOI: 10.1016/j.jad.2022.08.027 | Bacteria:  16S rRNA gene sequencing | Diagnosis:  Structured Clinical Interview for DSM-IV criteria  Symptoms:  Hamilton Depression Rating Scale (HAMD) and Young Manic Rating Scale (YMRS) | *Genus level*  ↑ Pesudomonadaceae in males with Bipolar Depression (BD) compared with Controls and MDD  ↑ Pesudomonadaceae in females with BD compared with Controls and MDD  ↑ Bacteroidaceae in females with MDD compared to Controls and BD  *OTUs*  ↑ 4 OTUs belonging to Bacteroidaceae (OTU1963, OTU1950, OTU1956, OTU1957) in MDD female  ↓ Lachnospiraceae OTU329 in male and female BD  ↑ 3 Lachnospiraceae OTUs (OTU36, OTU 761 and OTU 485) in female BD  2 Ruminococcaceae OTUs showed a separation enrichment in male and female BD  **Correlation analysis**  In MDD males, Pseudomonas OTU 385 positive correlated with YMRS score  In BD males, OTUs belonging to family Pseudomonas (positive: OTU385, OTU79) and Fusicatenibacter (negative: OTU329) significantly associated with HAMD  In MDD females, OTU 385 correlated with YMRS score, which showed consistent results in males  OTU901, belonging to family Romboutsia had a negative correlation with HAMD and YMRS scores Butyricicoccus OTU249 had a negative correlation with HAMD score in BD females vs MDD females | | | **Alpha and beta diversity**  ↓ Ace, Chao and Shannon in females with BD vs Controls |
| **36) Lim et al (2021)**  Cross-sectional associations with depressive scores in elderly(≥70yo), n= 176  DOI: 10.1093/gerona/glaa319 | Bacteria:  16S rRNA gene sequencing- Illumina MiSeq | Symptoms:  Geriatric Depression Scale (GDS) | *Genus level*  The GDS score showed a weak positive association with Clostridium and Campylobacter (coefficient = 0.0007 and 0.0008, respectively; FDR < 0.2) | | | **Alpha and beta diversity**  Significant association bet GDS score and composition distance ( Bray–Curtis distance, Adonis)  Significant negative association of GDS score with Shannon’s diversity and Pielou’s evenness |
| **37) Lin et al. (2017)**  Case-control study,n=20 (MDD, n=10; Controls, n=10)  Longitudinal intervention (MDD patients received Escitalopram) DOI: 10.1016/j.jad.2016.09.051 | Bacteria:  16S rRNA gene sequencing- Illumina MiSeq platform  qPCR to validate differentially abundant genera: Streptococcus, Clostridium XI, Prevotella and Klebsiella | Diagnosis:  DSM-IV-TR (text revision)  Symptoms:  Hamilton Depression Rating Scale (17-item version) | *Phylum level*  ↑ Firmicutes in MDD  ↓ Bacteroidetes in MDD  *Genus level*  ↑ *Clostridium XI, Klebsiella*, *Prevotella*, *Streptococcus* in MDD qPCR using genus specific primers validated ↑ *Prevotella* and *Klebsiella* but found no difference in the proportion of *Streptococcus* and *Clostridium XI* | | | **Alpha and beta diversity**  Species richness (ACE, Chao1) and richness/evenness (Shannon, Simpson) indices were estimated but not reported  MDD clustered separately from controls on PCoA (weighted UniFrac) at baseline and throughout escitalopram treatment |
| **38) Liśkiewicz et al. (2021)**  Intervention in MDD inpatients with Escitalopram,  n=16, DOI: 10.1016/j.pnpbp.2020.110076 | Bacteria:  16S rRNA gene 454 sequencing | Diagnosis:  ICD-10  Symptoms:  Hamilton Depression Rating Scale (24-item version) HDRS24 | The ASV and taxa abundance at the family level did not correlate significantly with HDRS24 at baseline  Significant correlations for the genus, order, class, and phylum taxonomic levels at baseline:  Positive correlation with Paraprevotella genus abundance  Negative correlations with the Clostridiales order in the class Clostridia in the phylum Firmicutes  Negative correlations the RF32 order in the class Alphaproteobacteria | | | **Alpha and beta diversity**  Alpha diversity did not correlate significantly with HDRS24 at baseline and did not differ bet severe and mild/moderate depression  PCoA did not reveal a clear separation bet patients with severe and mild/moderate depression |
| **39) Liu et al. (2022)**  Case-control, n=109  drug-naive MDD, n=66; Controls, n=43, DOI: 10.3389/fnins.2021.800764 | Bacteria:  16S rRNA gene sequencing- Illumina Novaseq PE250 | Diagnosis:  DSM-IV  Symptoms:  HAMD-17 | **LEfSe**  *Family level*  ↑ *Deinococcaceae* in MDD  ↑ *Bacteroidaceae, Turicibacteraceae, Clostridiaceae,* and *Barnesiellaceae* in Controls  *Genus level*  ↑*Deinococcus* and *Odoribacter* in MDD  ↑*Bacteroides, Alistipes, Turicibacter, Clostridium, Roseburia*, and *Enterobacter* in Controls | | | **Alpha and beta diversity**  ↓ Simpson index and Pielou’s evenness in MDD than Controls  Difference in composition (PCoA based on Jaccard dissimilarity, no statistical test) |
| **40) Liu et al. (2020)**  Case-control in young adults (18-25yo), n=90  MDD, n=43; Controls, n=47, DOI: 10.1016/j.bbi.2020.03.026 | Bacteria:  16S rRNA gene sequencing | Diagnosis:  Online PROMIS Depression scale, Phone screening with Structured Clinical Interview for DSM-5 Columbia-Suicide Severity Rating Scale and Self-Injurious Thoughts and Behaviour Interview, in-person assessment with PROMIS Depression questionnaire | *Phylum level*  ↑ Bacteroidetes in MDD  ↑ Firmicutes in Controls  *Class and order levels*  ↑ Clostridia (class) and Clostridiales (order) of Firmicutes in controls  ↑ Bacteroidia(class) and Bacteroidales (order) of Bacteroidetes in MDD  ↑ Rhodospirillales (order) Alphaproteobacteria (class) in controls  ↑ Gammaproteobacteria (class) in MDD group  *Family level*  ↑ Ruminococcaceae, Christensenellaceae, Bacteroidetes and uncultured family of Rhodospirillales in control ↑ Enterococcaceae (of the Bacilli-Lactobacillales lineage of Firmicutes) in MDD  *Genus level*  ↑ *Faecalibacterium, Subdoligranulum, [Eubacterium] coprostanoligenes group,CAG-352, Ruminococcus 1, Fusicatenibacter, Tyzzerella 3, [Eubacterium] ventriosum group, Barnesiella, Muribaculaceae uncultured bacterium, {Clostridiales} vadin BB60 family, Christensenellaceae R-7 group* and *Desulfovibrio* in control  ↑ *Flavonifractor, Sellimonas,* and *Enterococcus* in MDD | | | **Alpha and beta diversity**  No statistically significant difference in Observed ASVs metric and Shannon’s Diversity Index bet cases and controls  ↓ Faith’s Phylogenetic Diversity (relatedness of taxa) in MDD and was inversely related to the severity of depressive symptoms  Significant difference in composition bet groups (Bray-Curtis Dissimilarity, Unifrac Distance. No difference in Weighted Unifrac Distance bet groups |
| **41) Liu et al. (2016)** Case-control study, n=100 (IBS-D, n=40; Depression, n=15; Comorbid IBS-D and depression, n=25; Controls, n=20), DOI:10.1016/j.cgh.2016.05.033 | Bacteria:  16S rRNA gene 454 sequencing- Roche | Diagnosis:  MINI (DSM-IV)  Symptoms:  Self-Rating Depression Scale | *Phylum level*  ↑ Bacteroidetes in depression  ↓ Firmicutes in depression  *Genus level*  ↑ *Bacteroides, Dialister, Haemophilus, Paraprevotella, Prevotella, Veillonella* in depression  ↓ *Acetivibrio*, *Alistipes, Barnesiella, Bifidobacterium, Blautia, Butyricimonas, Clostridium IV*, *Clostridium XI, Clostridium sensu stricto, Comamonas, Coprococcus, Escherichia/Shigella, Faecalibacterium, Fusobacterium, Gemmiger,* *Lachnospiraceae incertae sedis, Megamonas, Mitsuokella, Odoribacter, Oscillibacter, Parabacteroides, Roseburia*, *Ruminococcus, Sutterella, Vampirovibrio* in depression | | | **Alpha and beta diversity**  ↓ Richness/ evenness (Shannon) in depression compared to controls  Did not analyse beta diversity |
| **42) Madan et al. (2020)**  Longitudinal associations with depression and anxiety severity in inpatients with serious mental illness, n= 111, DOI:10.1016/j.jad.2019.12.020 | Bacteria:  16S rRNA gene sequencing  Whole genome shotgun sequencing | Diagnosis:  Structured Clinical Interview for DSM-IV Disorders (SCID-I/II)  Symptoms:  Patient Health Questionnaire-9 (PHQ-9), Patient Health Questionnaire-Generalised anxiety disorder screener (GAD-7) | **LefSe-differential taxa associated with anxiety and depression severity shortly after admission**  *Taxa associated with depression at admission*  ↑ *Ruminococcus 1* in severe depression  ↑ *Flavonifractor* in moderate MDD  ↑ *Actinobacteria_phyla, Ruminococcaceae_UCG014, {Clostridiales} vadin BB60 family, Ruminococcaceae_UCG10* in mild MDD  *Species associated with depression at admission*  ↑ *Lachnospiraceae bacterium 1456FAA, Flavonifractor plautii* in severe MDD  ↑ *Streptococcus anginosus, Coprococcus catus, Clostridium symbiosum* in moderate MDD  ↑ *Bifidobacterium adolescentis, Alistipes, senegalensis, Oxalobacter formigenes* in mild MDD  *Taxa associated with anxiety at admission*  ↑ *Firmicutes, Clostridiales, Clostridia, Corynebacteriales, Actinobacteria (*class&phyla*), Eubacterium rectale group, Coprococcus1, RuminococcaceaeUCG010, Ruminococcus1, Lawsonella* in mild anxiety  *Species associated with anxiety at admission*  ↑ *Streptococcus anginosus, Lachnospiraceae bacterium 1456FAA* in severe anxiety  ↑ Lactococcus lactis, Eggerthella lenta, *Lachnospiraceae bacterium 1157FAA, Clostridiales bacterium 1747FAA, Eggerthella unclassified* in moderate anxiety  ↑ *Alistipes putredinis, Bifidobacterium adolescentis, Roseburia inulinivorans, Roseburia hominis, Eubacterium ventriosum, Coprococcus catus* in mild anxiety | | | **Alpha and beta diversity**  Depression and anxiety severity shortly after admission negatively associated with bacterial richness and alpha diversity |
| **43) Malan-Müller et al. (2023)**  Case-control, n=198  1 or more psychiatric symptoms(depression, state, and trait anxiety, PTSD), n=92; (Depressive symptoms, n=32; State anxiety symptoms, n=74; Trait anxiety symptoms, n=81, Symptoms of PTSD, n=16), DOI: 10.1080/19490976.2022.2162306 | Bacteria:  16S rRNA gene sequencing | Symptoms:  Depressive (Center for Epidemiologic Studies Depression), State-trait anxiety and depression inventory, PTSD Checklist for DSM-5 with Life Events Checklist for the Diagnostic and Statistical Manual of Mental Disorders, Fifth Edition | No significant differences in genus- or phylum-level gut microbiome community composition (measured by Aitchison distance, an Euclidean distance on clr-transformed data)  **Generalized linear models**  ↓ *Fusicatenibacter saccharivorans* in individuals with comorbid symptoms of PTSD + depression + state and trait anxiety (after correcting for main covariates) compared to individuals without these comorbid symptoms and to healthy controls  ↑ *Proteobacteria* in individuals with depressive symptoms compared to those without  ↓ *Synergistetes* in those with depressive symptoms compared to those without | | | **Alpha and beta diversity**  ↓ Simpson’s diversity in individuals with trait anxiety symptoms compared to those without and to controls |
| **44) Mason et al. (2020)**  Case-control study, n=70 (MDD, n=14; Anxiety, n=8, Comorbid anxiety and MDD, n=38; Controls, n=10), DOI: 10.1016/j.jad.2020.01.137 | Bacteria:  16S rRNA gene 454 sequencing- Roche  qPCR to quantify bacterial loads of Eubacteria, Enterobacteriaceae, Eubacterium rectale/Clostridium group (Clostridial cluster XIVa), Lactobacillus/Enterococcus, Bacteroides, Clostridium leptum group (Clostridial cluster IV) | Diagnosis:  SCID  Symptoms:  Quick Inventory of Depressive Symptoms  GAD-7  Snaith-Hamilton Pleasure Scale | **Taxonomic differences between participants with MDD only, anxiety only, comorbid anxiety and MDD, and controls on qPCR-targeted taxa** (Results in **bold** held after controlling for gender, age, race, and BMI)  ↓ Total bacteria in comorbidity compared to controls  ↓ ***Bacteroides*** in comorbidity compared to MDD only  ↓ ***Clostridium leptum* group** (Clostridial cluster IV) in MDD only and comorbid MDD/anxiety compared to controls | | | **Alpha and beta diversity**  No significant differences in richness/evenness (Shannon) and beta diversity (PCoA weighted UniFrac) between participants  No significant associations between richness/evenness or beta diversity and depression (QIDS-SR) or anxiety (GAD-7) symptoms  Hierarchical clustering using β-diversity (weighted UniFrac) defined two groups (Cluster 1 and Cluster 2). Cluster 2 had significantly higher anhedonia scores (Snaith-Hamilton Pleasure Scale) |
|  |  |  | **Correlation analysis**  Negative associations between:  - Total bacteria and anxiety severity (GAD-7)  ***- Eubacterium rectale/Clostridium* group (Clostridial cluster XIVa), *Clostridium leptum* group (Clostridial cluster IV)** and depression (QIDS-SR) and anxiety severity (GAD-7), controlling for gender, age, race and BMI | | |  |
|  |  |  | **Taxonomic differences between two clusters** (defined by distance-based clustering based on weighted UniFrac β-diversity) that differed significantly in terms of levels of anhedonia (based on Snaith-Hamilton Pleasure Scale): Cluster 1 = low anhedonia, Cluster 2 = high anhedonia group  *Order level*  ↑ Bacteroidales in Cluster 2  ↓ Clostridiales in Cluster 2  *Family level*  ↓ *Ruminococcaceae*, *Bifidobacteriaceae*, *Coriobacteriaceae*, *Christensenellaceae*, *Lachnospiraceae* in Cluster 2  *Genus level*  ↑ *Bacteroides* in Cluster 2  ↓ *Blautia*, *Collinsella*, *Gordonibacter*, *Roseburia* and *Bifidobacterium* in Cluster 2 | | |  |
| **45) Miyaho et al. (2022)**  Longitudinal age-specific associations in MDD, n=32  (Middle-Aged, n=18; Late-Life, n=14)  DOI: 10.3390/jpm12111827 | Bacteria:  16S rRNA gene sequencing | Diagnosis:  DSM-5, Hamilton Depression Scale (17 items) (HAM-D) and Hamilton Anxiety Scale (HAM-A) | **LEfSe at baseline**  *Family, Order and Class level*  ↑ *Lachnospiraceae, Clostridiales, Clostridia* in middle-aged vs late-life MDD  ↑ *Micrococcaceae, Micrococcales, FamlilyXIII, Pasteurellales, Pasteurellaceae, Bacillales, Staphylococcaceae, Enterobacteriales, Enterobacteriaceae, Bacilli* in late-life vas middle-aged MDD  *Genus level*  ↑ RuminococcaceaeUCG013, Staphylococcus, Raoultibacter, Coprococcus2, PrevotellaceaeNK3B31group, Megamonas, Intestinibacter, Eubacterium coprostanoligenesgroup, Klebsiella in late-life vas middle-aged MDD | | | **Alpha and beta diversity**  No significant differences in Chao1 index, or Shannon index at baseline |
| **46) Naseribafrouei et al. (2014)***  Case-control study, n=55 (Depression, n=37; Controls, n=18) | Bacteria:  16S rRNA gene sequencing- Illumina | Diagnosis:  Research criteria for ICD-10  Symptoms:  Montgomery-Åsberg  Depression Rating Scale | **False discovery rate corrected permutation testing for univariate differences in OTUs**  *Order level*  ↓ Bacteroidales associated with depression  No OTUs at any other level correlated significantly after false discovery correction. | | | **Alpha and beta diversity**  No significant differences in richness (number of OTUs) or richness/evenness (Simpson’s D) |
|  |  |  | **Multivariate PLS-DA to examine taxonomic correlations with depression using a regression model**  *Phylum level*  High representation of OTUs from Bacteroidetes correlating with depression  *Order level*  High representation of OTUs from Bacteroidales correlating with depression  *Family level*  Low representation of OTUs from *Lachnospiraceae* correlating with depression  *Genus level*  High representation of correlating OTUs from clades within *Alistipes* and *Oscillibacter* but both positive and negative associations observed for different OTUs within each clade.  No species identified – OTUs reported by number ID. | | | Multivariate PLS-DA analysis: good sensitivity and specificity (100% of the classified depressed patients and 97% of the classified non-depressed).  Model was dependent on OTU-level specificity (binning data to genus-level gave poor classification), but OTUs were only identified by OTU number |
| **47) Radjabzadeh et al. (2022)**  Gut microbiome-wide association with depressive symptoms, n=2593 [Rotterdam, n=1,054; HELIUS (validation), n=1,539]  DOI: 10.1038/s41467-022-34502-3 | Bacteria:  16S rRNA gene sequencing- Illumina MiSeq | Symptoms:  Rotterdam: 20-item version of the Center for Epidemiological Studies-Depression  HELIUS: Patient Health Questionnaire | **Significantly associated taxa with depressive symptom levels in both Rotterdam and HELIUS cohorts and in meta-analysis**  *Negative*  *RuminococcaceaeUCG005.id.11363, Ruminococcaceae.id.2050,* *RuminococcaceaeUCG002.id.11360, RuminococcaceaeUCG003.id.11361,Coprococcus3.id.11303, LachnospiraceaeUCG001.id.11321,*  *Ruminococcusgauvreauiigroup.id.11342, Eubacteriumventriosumgroup.id.11341,* *Subdoligranulum.id.2070*  *Positive*  *Lachnoclostridium.id.11308, Eggerthella.id.819, Hungatella.id.11306, Sellimonas.id.14369*  **Predictors of depressive symptoms-Random forest with Rotterdam as training and HELIUS as testing**  *RuminococcaceaeUCG005* most important genus in predicting depressive symptoms  *ChristensenellaceaeR7group, Lachnoclostridium, Eggerthella, Sellimonas, Hungatella, Roseburia, Streptococcus, Bacteroides, Anaerotruncus, Dorea, Blautia, Veillonella, Desulfovibrio, Anaerostipes* and *Bifidobacterium*  **Mendelian Randomization analysis identifies a causal link between MDD and Eggerthella** | | | **Alpha and beta diversity**  Shannon was negatively associated with depressive symptoms in both Rotterdam and HELIUS cohorts  Beta diversity showed significant association with depressive symptoms in RS but not in the HELIUS |
| **48) Renson et al. (2020)**  Wisconsin Longitudinal Study, n=313 (Depression and anxiety, n ​= ​236)  DOI: 10.1016/j.bbih.2020.100155 | Bacteria:  16S rRNA gene sequencing- Illumina MiSeq | Symptoms:  Depression: Center for Epidemiological Studies – Depression Scale (CES-D)  Anxiety: Spielberger Anxiety Index | **Significantly differential abundant taxa (CES-D)-genus and species level**  *Negative*  *Cloacibacillus, Rothia mucilaginosa, Pseudoramibacter_Eubacterium, Lachnobacterium, Eggerthella lenta*, *Blautia obeum, Clostridium hathewayi, Eggerthella*  *Positive*  *Desulfovibrio D16, Prevotella, Clostridium colinum, Prevotella, Campylobacter, Mogibacterium, Acidaminococcus, Megamonas, Clostridium butyricum, Prevotella copri, Pseudoramibacter_Eubacterium, Olsenella*  **Significantly differential abundant taxa (anxiety)-genus and species level**  *Negative*  *Megamonas, Cloacibacillus, Clostridium, Clostridium ramosum, Clostridium spiroforme,*  *Streptococcus sobrinus, Fusobacterium, Eubacterium, [Eubacterium]dolichum*  *Positive*  *Bacteroides plebeius, Desulfovibrio D168, Clostridium colinum, Clostridium butyricum, Coprobacillus cateniformis, Streptococcus luteciae, Mogibacterium, Campylobacter, Veillonella, Corynebacterium* | | | **Alpha and beta diversity**  Did not analyse |
| **49) Ritchie et al. (2023)**  Exploratory study,  Gut microbiome differences bet MDD with and without anxious distress, n=117  [MDD with (n=63) and without (n=54) anxious distress]  DOI:10.1016/j.jad.2022.10.001 | Bacteria:  16S rRNA gene sequencing- Illumina | Diagnosis:  Structured clinical interview for the DSM-5-RV  Symptoms:  Severity of depression (BDI-II), generalized anxiety (GAD-7), and stress (PSS) | **Significantly differential abundant taxa bet MDD with and without anxious distress**  *Genus level*  ↓ Candidatus Amoebophilus, Catenibacterium**, Limnobacter  ↑ Faecalitalea*, Turicibacter, Clostridium_X, Megamonas, Veillonella, Megasphaera**, Cetobacterium*, Fusobacterium**, Thermodesulfovibrio, Serratia*, Akkermansia, Escherichia*  *Species level*  ↑ Eggerthella sinensis*, Bacteroides coprocola**, Parabacteroides johnsonii*, Eubacterium cylindroides*  ↑ Turicibacter sanguinis**, Clostridium fallax, Sarcina maxima, Clostridium cadaveris, Megamonas funiformis, Megasphaera hominis**, Cetobacterium ceti*, Fusobacterium gonidiaformans, Thermodesulfovibrio thiophilus, Escherichia coli*, Escherichia albertii*, Serratia entomophila*  ↓ Prevotella stercorea**, Catenibacterium mitsuokai  *p <.01 **p<.001 | | | **Alpha and beta diversity**  No significant group differences in α-diversity (Shannon's diversity Index; Simpson Index), richness (ACE; Chao1), (Pielou's) evenness, or beta diversity (Bray-Curtis dissimilarity index and weighted UniFrac distance)  **Other**  Suggestive correlations bet some species/genera and symptoms; none reached significance after allowing for multiple comparisons |
| **50) Naudé et al. (2019)**  Cross-sectional associations, n=84, Depression and Post-traumatic stress disorder symptoms, DOI: 10.1017/neu.2019.43 | Bacteria:  16S rRNA gene sequencing- Illumina MiSeq platform | Symptoms:  Beck Depression Inventory-II  Modified PTSD Symptom Scale | No significant association between pre-pregnancy depression and maternal faecal bacterial diversity at birth of child | | | **Alpha and beta diversity**  Prenatal psychological measures not associated with maternal richness/evenness (Shannon)  No clustering patterns for β-diversity (W metric) |
| **51) Rong et al. (2019)** Case-control study, n = 91 (MDD, n=22; Bipolar Disorder, n=30; Controls, n=30), DOI: 10.1016/j.jpsychires.2019.03.017 | Shotgun metagenomics- Illumina HiSeq2500 | Diagnosis:  DSM-5, with current Hamilton Depression Rating Scale-17 score of >17 during a depressive episode  Symptoms:  Hamilton Depression Rating Scale  Hamilton Anxiety Rating Scale  Mood Disorder Questionnaire  Hypomania Checklist (HCL-32) | *Phylum level*  ↑ Actinobacteria, Firmicutes in MDD  ↓ Bacteroidetes in MDD  *Order level*  ↑ Selemonadales in MDD  ↓ Alteromonadales in MDD  *Family level*  ↑ *Bifidobacteriaceae, Coriobacteriaceae, Desulfovibrionaceae, Enterobacteriaceae, Micrococcineae, Ruminococcaceae, Veillonellaceae* in MDD  ↓ *Chitinophagaceae, Leptotrichiaceae, Pasteurellaceae* in MDD  *Genus level*  ↑ *Acidaminococcus,* *Bifidobacterium, Cellulosilyticum, Clostridium, Desulfitobacterium, Desulfotomaculum, Desulfovibrio, Eggerthella, Enterococcus, Ethanoligenes, Heliobacterium, Lactobacillus, Megasphaera, Oscillibacter, Selenomonas, Sphaerochaeta, Streptococcus, Treponema* in MDD  ↓ *Bacteroides, Haemophilus, Odoribacter, Paludibacter, Porphyromonas, Tannerella* in MDD  ↔ *Veillonella,* (unable to decipher direction from figure nor supplemental)  *Species level*  230 species and subspecies showed significant changes between MDD and Controls. Top 20:  ↑ *Acidaminococcus intestini RyC-MR95, Akkermansia muciniphila ATCC BAA-835, Bifidobacterium, Bifidobacterium adolescentis ATCC 15703, Bifidobacterium dentium Bd1, Bifidobacterium longum, Clostridium saccharolyticum WM1, Escherichia coli, Eubacterium rectale,* *Eubacterium rectale ATCC 33656,* *Megasphaera elsdenii DSM 20460, Oscillibacter valericigenes Sjm18-20, Prevotella denticola F0289, Prevotella intermedia 17, Prevotella melaninogenica ATCC 25845, Selenomonas ruminantium subsp. lactilytica TAM6421, Selenomonas sputigena ATCC 35185* in MDD  ↓ *Haemophilus parainfluenzae* T3T1 in MDD | | | **Alpha and beta diversity**  ↓ Richness in MDD compared to controls (Chao1)  ↓ Richness/evenness in a novel indicator (G_m_ coefficient) but no significant differences in other indices (Shannon, Inverse Simpson) in MDD compared to controls  No significant difference between groups on PCoA (Bray-Curtis)  Positive correlation between anxiety symptoms (HAM-A) and alpha diversity (G_m_ coefficient)  **Other**  Refer to paper for comprehensive metabolomic analyses |
| **52) Šik Novak et al. (2022)**  Longitudinal associations with psychological measures (depression, anxiety and stress) to identify the effect of COVID-19 lockdown, healthy adults, n=38, DOI:  10.1080/10253890.2022.2082280 | Bacteria:  16S rRNA gene sequencing | Symptoms:  State-Trait Anxiety Inventory (STAIX-1), The Center for Epidemiologic Studies Depression Scale (CES-D) and The Perceived Stress Scale (PSS), The Positive and Negative Affect Schedule (PANAS) and Body Dissatisfaction (BD) | Post-lockdown, Proteobacteria abundance significantly increased  **Correlation analysis between psychological measures and gut microbiota composition**  Moderate positive correlation bet change in Bacteroidetes abundance and depression during lockdown Moderate negative correlation bet change in Firmicutes abundance and depression during lockdown  Alistipes abundance post-lockdown moderately positively correlated with anxiety during lockdown and with negative affect during lockdown  Weak negative correlation bet Bacteroides abundance post-lockdown and positive affect during lockdown  **Partial correlations bet psychological measures and bacterial abundance controlling for gender and diet**  Bacteroidetes abundance post-lockdown moderately positively correlated with depression during lockdown and negatively with positive affect during lockdown  Firmicutes/Bacteroidetes ratio post-lockdown was moderately negatively correlated with depression during lockdown | | | **Alpha and beta diversity**  Post-lockdown alpha diversity significantly decreased |
| **53) Stanislawski et al. (2021)**  Cross-sectional observational study of US military Veterans, n= 331, DOI: 10.1016/j.ahj.2021.05.002 | Bacteria:  16S rRNA gene sequencing- Illumina MiSeq | Diagnosis:  Structured Clinical Interview for the Diagnostics and Statistical Manual of Mental Disorders (SCID) Version 5 (DSM-5)  Symptoms:  Beck Depression Inventory (BDI) | **Taxonomic differences (FDR < 0.05) using analysis of composition of microbiomes (ANCOM)**  Presence of severe depressive symptoms were inversely associated with Ruminococcus1  Presence of bipolar disorders was directly associated with Bacteroides  Presence of anxiety disorders was directly correlated with Blautia | | | **Alpha and beta diversity**  Bipolar disorders and depressive symptoms (BDI) correlated with unweighted UniFrac (β-diversity) |
| **54) Stevens, Pepine et al. (2021)**  Case-control, n=54 (Hypertension, n=18; Depression, n=7; Depression&  Hypertension, n=8; Controls, n=21), DOI: 10.1016/j.ahj.2021.05.002 | Whole metagenome shotgun sequencing | Diagnosis:  DSM-5 | **Gut microbial taxa ecology differences**  The 4 groups were isolated based on hierarchical cluster dissimilarities (no statistical test)  **Dominant prevalent taxa using denoising machine-learning pipeline (statistical significances were validated by Mann-Whitney, Bray-Curtis dissimilarity, Spearman's Rho, effect sizes, and Dirichlet Monte-Carlo simulations)**  *Bifidobacterium_longum, Coprococcus_catus***,** *Escherichia_coli***,** *Roseburia_intestinalis* in Controls  *Clostridium_symbiosum, Lachnospiraceae_bacterium_3_1_46FAA, Veillonella_unclassified* in depression | | | **Alpha and beta diversity**  Not analysed |
| **55) Stevens, Roesch et al. (2021)**  Case-control, n=40 (Depression, n=20; Controls, n=20), DOI: 10.1038/s41380-020-0652-5 | Bacteria:  16S rRNA gene sequencing- Illumina MiSeq | Diagnosis:  DSM-IV | **ADLEx2/PIME analysis of ASVs for depression and controls (Bonferroni)**  *Acidaminococcaceae_Phascolarctobacterium_faecium, Tannerellaceae_Parabacteroides_merdae, Bacteroidaceae_Bacteroides_null_5, Enterobacteriaceae_Escherichia/Shigella_null_1, Lachnospiraceae_Lachnoclostridium_null_8, Bacteroidaceae_Bacteroides_stercoris_1,*  *Ruminococcaceae_Flavonifractor_plautii_1, Lachnospiraceae_Lachnoclostridium_null_5*  *Ruminococcaceae_Ruminiclostridium_9_null_1, Lachnospiraceae_null_null_10, Ruminococcaceae_Phocea_massiliensis, Ruminococcaceae_null_null_10, Saccharimonadaceae_null_null_1, Lachnospiraceae_null_null_12, Erysipelotrichaceae_Holdemania_filiformis* in Depression  *Ruminococcaceae_Faecalibacterium_CM04-06, Lachnospiraceae_Lachnospiraceae_NK4A136_group_null_1, Lachnospiraceae_Lachnospiraceae_UCG-001_null_20, Lachnospiraceae_Lachnoclostridium_null_1, Bacteroidaceae_Bacteroides_vulgatus_2, Ruminococcaceae_Ruminococcaceae_UCG-003_null_1, Lachnospiraceae_Roseburia_intestinalis, Ruminococcaceae_Ruminococcaceae_UCG-002_null_2, Lachnospiraceae_Lachnospiraceae_NK4A136_group_null_4, Bacteroidaceae_Bacteroides_null_3*  *Lachnospiraceae_Lachnoclostridium_null_3, Lachnospiraceae_Lachnospiraceae_ND3007_group_null_1,*  *Lachnospiraceae_Lachnospira_pectinoschiza_2, Lachnospiraceae_Agathobacter_null_2, Lachnospiraceae_Lachnospiraceae_NK4A136_group_null_3, Lachnospiraceae_null_null_15, Bacteroidaceae_Bacteroides_null_6, Lachnospiraceae_Lachnospiraceae_UCG-001_null_21, Lachnospiraceae_null_null_17, Lachnospiraceae_Lachnospira_null_2, Lachnospiraceae_null_null_8,*  *Lachnospiraceae_CAG-56_null_1, Lachnospiraceae_Lachnospiraceae_UCG-001_null_42,*  *Ruminococcaceae_Faecalibacterium_null_2* in Controls | | | **Alpha and beta diversity**  Did not analyse |
| **56) Sun et al. (2022)**  Case-control, n=60 (MDD, n=31; Controls, n=29), DOI: 10.1111/pcn.13368 | Bacteria:  16S rRNA gene sequencing | Diagnosis:  DSM-IV using the M.I.N.I.  Symptoms:  Hamilton Depression (HAMD) score | **LEfSe**  *Order class and family level*  ↑ Bacteroidia, Bacteroidales, Bacteroidetes, Bacteroidaceae, Ruminococcaceae, Clostridiaceae, Clostridiaceae, [Barnesiellaceae], Turicibacterales, Turicibacteraceae in Controls  ↑ Deinococcaceae, Deinococcale in MDD  *Genus level*  ↑ Bacteroides, Ruminococcus, Clostridium, Barnesiella, Turicibacter in Controls  ↑ Deinococcus in MDD  **Negative correlations with HAMD**  Turicibacter, Clostridium, Bacteroides, Clostridiaceae, Turicibacteraceae, [Barnesiellaceae], Bacteroidaceae, Turicibacterales, Bacteroidia, Bacteroidales, Bacteroidetes | | | **Alpha and beta diversity**  ↑ Simpson and Pielou evenness in Controls than MDD  Differences in β-diversity (nonmetric multidimensional scaling analysis) |
| **57) Szczesniak et al. (2016)***  Case-control, n=51 (Depression, n=34; Controls, n=17), DOI:10.1179/1476830515Y.0000000007 | Bacteria:  16S rRNA gene sequencing- Illumina | Diagnosis:  Research criteria for ICD-10  Symptoms:  Montgomery-Åsberg  Depression Rating Scale | **Group specific microbiota findings previously reported in Naseribafrouei et al. (2014)**  Positive correlations between OTUs belonging to: *Alistipes* (OTU7)*, Bacteroides dorei* (OTU6), *Faecalibacterium* (OTU50)*, Oscillibacter* (OTU16), *Ruminococcus* (OTU11) and depression (MADRS)  Negative correlations between OTUs belonging to: *Bacteroides dorei* (OTU4)*, Bacteroides uniformis* (OTU8), *Faecalibacterium* (OTU12), *Ruminococcus* (OTU25) and depression (MADRS) | | | **Alpha and beta diversity**  Did not analyse  **Other**  Propionic, isobutyric, and isovaleric acids were associated with depression  Cortisol not different, but associated with volatile fatty acids |
| **58) Taylor et al. (2019)**  Cross-sectional associations with symptom measures , n=133 (males, n=60; females, n=73), DOI:  10.1080/1028415X.2019.1582578 | Bacteria:  16S rRNA gene sequencing | Symptoms:  42-item Depression, Anxiety, and Stress Scale (DASS-42) | **Associations between DASS-42 scores in whole sample (both male and female) after controlling for age and BMI**  (Results in **bold** after controlling for dietary fibre intake (in addition to age and BMI))  Positive correlations between:  - *Enterobacteriaceae* and depression - ***Peptostreptococcaeae* and anxiety**  Negative correlations between:  *-* ***Porphyromonadaceae*** and anxiety, **and total DASS**  ***- Anaerostipes* and depression, and total DASS**  - ***Parabacteroides*** and anxiety, **and total DASS** | | | **Alpha and beta diversity**  No difference in richness/evenness (Shannon) or phylogenetic diversity between males and females, but no reported analysis with reference to anxiety or depression symptoms  No difference in β-diversity (weighted UniFrac) between males and females, but no reported analysis with reference to anxiety or depression symptoms  **Other**  Also examined stress subscales not reported herein |
|  |  |  | Males | | Females |  |
|  |  |  | Positive correlations between:  - ***Erysipelotrichaceae and* depression**  **-** ***Peptostreptococcaceae* and anxiety**  - ***Roseburia* and depression** *- Phascolarctobacterium* and anxiety  Negative correlations between:  - ***Lactobacillaceae* and depression**  ***- Rikenellaceae* and depression**  - ***Dorea*** **and** **depression, and anxiety**  **- *Lactobacillus* and depression**  ***-*** *Ruminococcus* and depression  - ***Blautia* and anxiety** | | Positive correlations between:  - ***Coriobacteriaceae* and depression**  *- Lactobacillaceae* and anxiety  ***- Collinsella* and depression**  - ***Dialister* and depression, and anxiety, and total DASS**  *- Lactobacillus* and anxiety  **- *Paraprevotella* and anxiety,** and depression  Negative correlations between:  - **Proteobacteria and total DASS**  - ***Erysipelotrichaceae* and depression, and total DASS**  ***- Bifidobacteriaceae* and anxiety**  - ***Peptococcaceae* and anxiety**  *- Porphyromonadaceae* and anxiety  - *Anaerostipes* and depression  *-* ***Bifidobacterium* and anxiety**  **-** *Parabacteroides* and anxiety  - ***Eubacterium* and total DASS** |  |
| **59) Tsai et al. (2022)**  Case-control, n=53  [Late-life depression (LLD) , n=36; Controls, n=17], DOI: 10.3389/fnagi.2022.885393 | Bacteria:  16S rRNA gene sequencing | Diagnosis:  DSM-IV-TR  Symptoms:  17-item Hamilton Depression Rating Scale (HAMD) | **LEfSe**  *Phylum level*  ↑ *Verrucomicrobiota and Patescibacteria,*in LLD  *Class level*  ↑ *Verrucomicrobiae, Alphaproteobacteria, and Saccharimonadia* in LLD  *Order level*  ↑ *Verrucomicrobiales, Pasterurellales, Saccharimonadales, and Micrococcales* in LLD  ↑ *Flavobacteriales, Christinsenellales,* and *Clostridia_UCG014* in Controls  *Family level*  ↑ *Akkermansiaceae, Burkholderiaceae, Pasteurellaceae, Micrococcaceae Leuconostocaceae* and *Atopobiaceae* in LLD  ↑ *Flavobacteriaceae, vadinBE97,Christinsenellaceae, Oscillospiraceae* in Controls  *Genus level*  ↑ *Eggerthella, Blautia, Olsenella, Haemophilus, Enterobacter, Burkholderia,* and *Rothia* in LLD  ↑ *Sanguibacteroides, Eubacterium xylanophilum group, Christinsenellaceae R7group, NK4A214group, Ruminococcus gauvreauii group, UCG003, Lachnospiraceae UCG001, CAG56, Eubacterium ventriosum group, UCG002, Lachnospiraceae ND3007 group, Alistipes, Eubacterium ruminantium group* in Controls  **Correlations with HAMD**  *Positive: Enterobacter and Burkholderia in LLD*  *Negative: Sanguibacteroides in Controls* | | | **Alpha and beta diversity**  ↑ within-sample (α) phylogenetic diversity analysis (by FPD) in Controls vs LLD  Significant difference in unweighted UniFrac distance bet groups |
| **60) Valles-Colomer et al. (2019)**  Case-control with two independent studies (Flemish Gut Flora Project [FGFP], n=1054; Dutch LifeLines DEEP [LLD], n=1063) (Depression and Controls), DOI: 10.1038/s41564-018-0337-x | FGFP:  Bacteria:  16S rRNA gene sequencing- Illumina HiSeq2500  Shotgun metagenomics- Illumina HiSeq2500  As described in Tigchelaar et al. (2015),  LLD:  Bacteria:  16S rRNA gene sequencing – Illumina MiSeq platform  Shotgun metagenomics- Illumina HiSeq2500 | Diagnosis:  General practitioner-reported depression diagnosis in FGFP  Self-reported history of depression in LLD cohort | **After false discovery rate correction:**  ↓ ***Coprococcus* and *Dialister*** in depression (after partialling out the effects of antidepressant medication), validated in in both independent datasets (FGFP and LLD)  ↓ *Butyricicoccus,* *Fusicatenibacter* in depression, but did not remain significant after controlling for antidepressant treatment  **Replications of previous published case-control studies in FGFP cohort (not significant after controlling for antidepressant treatment):**  ↑ *Holdemania, Lactobacillus, Parabacteroides, Phascolarctobacterium* in depression  ↓ *Coprococcus, Dialister, Faecalibacterium, Turicibacter* in depression | | | **Alpha and beta diversity**  Depression included as explanatory variable for microbiome variation (Aitchison distance) in stepwise distance-based redundancy analysis, with explanatory power *R*^2^ <.25.  Depression was the most prevalent psychiatric disorder in FGFP cohort (11.5%) explaining 0.13% of microbiota compositional variation.  **Other**  Also examined quality of life and inferred microbial metabolism; however, results not reported herein  ↑ prevalence of *Bacteroides* enterotype 2 samples in depression compared to no-depression (26 versus 13%) in the FGFP data set (dirichlet multinomial mixtures) |
| **61) Vinberg et al. (2019)**  Case-control twin study, n=128 comparing affected twins (remission with either unipolar or bipolar), n=71; unaffected high-risk twins with a co-twin history of affective disorder, n=32; low-risk twins with no personal or family history of affective disorder, n=25, DOI: 10.1111/acps.12976 | Bacteria:  16S rRNA gene sequencing- Illumina MiSeq | Diagnosis:  ICD, 8th revision and 10th revision confirmed by the SCAN interview  Symptoms:  Hamilton Depression Rating Scale-17 items | **Logistic regression with lasso regularization**  *Phylum level*  No variables selected, suggesting this level did not contain information for discrimination  *Class, order, family, and genus levels*  A single taxonomic predictive variable was selected: “Firmicutes_unclassified,” (set of 27 OTUs identified as Firmicutes but not identified at more detailed resolution levels)  ↓ “Firmicutes_unclassified,” associated with disease  *OTU level*  A single OTU selected: OTU “103_r57651_1”, an uncultured bacterium from the *Christensenellaceae* R-7 group. This OTU was often absent from the affected (absent in 58%) and high-risk group (50%) compared to the low-risk group (12%), and was significantly decreased in unipolar affected and high-risk twins compared to the low-risk group. However, this difference did not hold when comparing only discordant twin pairs and did not correlate with differences within twin pairs in depression scores (HAM-D). | | | **Alpha and beta diversity**  ↓ Richness in depression compared to controls (number of observed OTUs)  Non-significant trend toward ↓ richness/evenness (Shannon, p = .07)  Trend for ↓ richness in high-risk group compared to low-risk group (p = .06)  No significant difference between groups on PCoA/ PERMANOVA (generalized UniFrac) |
| **62) Yang et al. (2020)**  Case-control, n= 311 (MDD, n=156; Controls, n=155) DOI:10.1126/sciadv.aba8555 | Whole-genome shotgun metagenomics | Diagnosis:  DSM-IV  Symptoms:  HAMD-17, 16-item Quick Inventory of Depressive Symptomatology–Self-Report | **Differential bacteria species between MDD and HC groups**  *Family level*  ↑ Bacteroidaceae, Bifidobacteriaceae, Veillonellaceae, unclassified_o__Bacteroidales, Acidaminococcaceae  Porphyromonadaceae, Eubacteriaceae in MDD  ↑ Lachnospiraceae, Clostridiaceae, Eubacteriaceae, Ruminococcaceae, Eggerthellaceae, unclassified_p__Firmicutes, unclassified_c__Bacilli, Enterococcaceae, Oscillospiraceae, Enterobacteriaceae in Controls  *Genus level*  ↑ *Bacteroides, Bifidobacterium, Veillonella, unclassified_o__Bacteroidales, Phascolarctobacterium, Parabacteroides, Eubacterium* in MDD  *Blautia, Clostridium, Eubacterium, Faecalibacterium, Coprococcus, Anaerostipes, Adlercreutzia, unclassified_p__Firmicutes, unclassified_c__Bacilli, Ruminococcus, Dorea, Enterococcus, Subdoligranulum,*  *Oscillibacter, Citrobacter, Klebsiella* in Controls  *Species level*  ↑ *Bacteroides_thetaiotaomicron, Bifidobacterium_longum,Veillonella_sp._CAG:933, unclassified_o__Bacteroidales, Bacteroides_stercoris, Bacteroides_stercoris_CAG:120, unclassified_g__Bacteroides, Bacteroides_fragilis, unclassified_g__Bifidobacterium, Phascolarctobacterium_sp._CAG:207, Bacteroides_massiliensis, Bacteroides_dorei, Bacteroides_vulgatus, Bacteroides_ovatus, Parabacteroides_distasonis, Eubacterium_sp._CAG:146, Eubacterium_sp._CAG:180, Bacteroides_eggerthii*  in MDD  *Blautia_obeum, Clostridium_sp._CAG:217, Eubacterium_sp._CAG:202, Faecalibacterium_sp._CAG:74, Coprococcus_eutactus, Anaerostipes_hadrus, Adlercreutzia_equolifaciens, Firmicutes_bacterium_CAG:227, unclassified_c__Bacilli, Eubacterium_sp._CAG:156, Blautia_sp._GD8, Ruminococcus_sp._5_1_39BFAA, Dorea_sp._CAG:105, [Eubacterium]_hallii, Enterococcus_faecalis,Eubacterium_hallii_CAG:12, Blautia_wexlerae, Faecalibacterium_prausnitzii, Subdoligranulum_variabile, Blautia_sp._Marseille-P2398, Oscillibacter_sp._ER4, Citrobacter_freundii, unclassified_g__Klebsiella, Clostridium_sp._CAG:510, Clostridium_sp._CAG:62, Eubacterium_ventriosum, Klebsiella_pneumoniae, Firmicutes_bacterium_CAG:41, Blautia_sp._CAG:237* in Controls | | | **Alpha and beta diversity**  No significant difference among a indexes bet the two groups  Significant difference in Bray-Curtis distance bet the 2 groups (PCoA, PERMANOVA) |
| **63) Ye et al. (2021)**  Case-control, n=54 (MDD, n=26; Controls, n=28), Intervention with vortioxetine, DOI: 10.3389/fpsyt.2021.641491 | Bacteria:  16S rRNA gene sequencing-pyrosequencing | Diagnosis:  Clinical interviews DSM-IV axis I criteria  HAMD-17 ≥ 24  Symptoms:  HAMD-17 | **LEfSe**  *Phylum level*  ↑ Proteobacteria and Acidobacteria in MDD  ↑ Firmicutes in Controls  *Genus level*  ↑ *Dialister, Parabacteroides, Aquisphaera,* and *Bacillus* in MDD  ↑ *Romboutsia, Phascolarctobacterium, Roseburia, Prevotella-2,* and *Lachnospira* in Controls | | | **Alpha and beta diversity**  ↑ Chao1 and Shannon in MDD  Significant differences in unweighted UniFrac bet groups |
| **64) Zhang et al. (2022)**  Case-control, n=81 (MDD, n=36; Controls, n=45), DOI:10.1016/j.jpsychores.2022.110787 | Bacteria:  16S rRNA gene sequencing | Diagnosis:  ICD-10  HAM-D > 17  Symptoms:  Hamilton Anxiety Scale (HAMA), and Hamilton Depression Scale (HAMD) | **Differential abundance of operational taxonomic units (OTUs) bet MDD and Controls using metastates (after FDR correction)**  ↑ OTU255 and OTU363 (Firmicutes_unclassified), OTU244 (Firmicutes_Intestinimonas), OTU80 (Firmicutes_Flavonifractor), OTU221 (Firmicutes_Lachnoclostridium), OTU725 (Bacteroidetes_Bacteroides), OTU542 (Firmicutes_Lachnoclostridium) in MDD  ↓ OTU423 and OTU502 (Firmicutes_unclassified) in MDD  **Spearman’s and partial correlation between the differentially abundant taxa (after FDR correction and sex adjustment)**  OTU255 (unclassified), OUT363 (unclassified), OTU244 (Intestinimonas), OTU542 (Lachnoclostridium), OTU221 (Lachnoclostridium), OTU725 (Bacteroides) and OTU80 (Flavonifractor) were positively correlated with HAMD and HAMA  OTU423 (unclassified) and OTU502 (unclassified) were negatively correlated with FSS, ESS, HAMD and HAMA | | | **Alpha and beta diversity**  No significant difference in Chao, ACE, Shannon, and Simpson bet MDD and Controls  Alpha diversity was not significantly associated with HAMA, or HAMD scores in MDD or Controls |
| **65) Zhang et al. (2021)**  Case-control, n=81 (MDD, n=36; Controls, n=45), DOI: 10.3389/fpsyt.2021.645045 | Bacteria:  16S rRNA gene sequencing | Diagnosis:  ICD-10  HAM-D > 17  Symptoms:  Hamilton Depression Scale (HAMD) | **Differential abundance of taxa bet MDD and Controls (after FDR correction)**  *Genus level*  ↑ *Flavonifractor, Alloprevotella, Parabacteroides, Hungatella, Bacteroides Tyzzerella, Intestinimonas* and *Eisenbergiella* in MDD  *Species level*  ↑ *Subdoligranulum_sp._4_3_54A2FAA,* and *Eggerthella_lenta,* in MDD  ↓ *uncultured_bacterium,* and *uncultured_Veillonellaceae_bacterium* in MDD  **LEfSe**  ↑ *Bacteroidaceae, Bacteroides,* and *uncultured_Mesorhizobium_sp.* in Controls  ↑ uncultured_bacterium, Prevotellaceae, and Prevotella in MDD  **Correlations with HAMD in Controls (after FDR correction-in MDD no stat. sign. corr. after FDR)**  *Positive: Haemophilus* (genus) and *Haemophilus_parainfluenzae* (species) with HAM-D | | | **Alpha and beta diversity**  No difference in Chao, ACE, Shannon, and Simpson bet MDD and Controls  Statistically significant difference in β diversity (unweighted & weighted UniFrac distance) |
| **66) Zhang, Hou et al. (2022)**  Cross-sectional associations, MDD, n= 39 [Depression or anxiety subgroups (mild, moderate, severe) based on the HAMD and HAMA], DOI:10.1016/j.neuroscience.2022.06.024 | Bacteria:  16S rRNA gene sequencing | Diagnosis:  DSM-5  Symptoms:  Hamilton depression scale-17 (HAMD-17) and Hamilton anxiety scale-14 (HAMA-14) | **Linear regression analysis**  *Genus level*  *Akkermansia, Phascolarctobacterium, Coprococcus* and *Streptococcus* positively correlated with HAMD *Bacteroides pleibeius* and *Bacteroides fragilis* had negative correlations with HAMD  *Faecalibacterium, Veillonella, Veillonella parvula* and *Streptococcus* had positive correlations with HAMA  *Clostridium* and *Bacteroides fragilis* had negative correlations with HAMA  **Discriminative genera among MDD subgroups based on HAMD scores**  ↑ *Akkermansia* and *Phascolarctobacterium* genera in depression  **Discriminative genera among MDD subgroups based on HAMA scores**  ↑ *Akkermansia*, *Coprococcus* and *Streptococcus* genera and ↑ *Akkermansia* *muciniphila* and *Streptococcus* *infantis* in anxiety  **Random forest analysis of the major taxonomic OTUs among the subgroups based on HAMA**  *Streptococcus* and *Streptococcus infantis* were among the top 3 most discriminative genera and species | | | **Alpha and beta diversity**  No significant differences in Chao, Shannon’s and Simpson’s bet subgroups  No separation of the three groups segregated in the PCA plots of Aitchison’s distance by either the HAMD or HAMA score |
| **67) Zhang, Zhang et al. (2022)**  Case-control, n=90 (MDD, n=53; Controls, n=37), MDD subgroup with childhood maltreatment (CTQ1), n=35,DOI:10.3389/fnins.2022.926450 | Bacteria:  16S rRNA gene sequencing | Diagnosis:  DSM-IV  Symptoms:  HAMD-24, Childhood Trauma Questionnaire-Short Form (CTQ) | **LefSe**  *Family level*  ↑ Bifidobacteriaceae in CTQ1 group, and Enterococcaceae in CTQ0 group  ↑ Bacteroidaceae, Ruminococcaceae, Veillonellaceae, Rikenellaceae, and Porphyromonadaceae in Controls  *Genus level*  ↑ *Bifidobacterium* in CTQ1 group, Blautia and Enterococcus in CTQ0 group  ↑ *Bacteroides, Roseburia, Faecalibacterium, Megamonas, Gemmiger, Dialister, Ruminococcus,* *Parabacteroides, Alistipes, Phascolarctobacterium*, and *Oscillospira* in Controls  **Correlation between the different gut microbiota and clinical parameters**  *-Blautia* and *Bifidobacterium* were significantly positively correlated with HAMD-24 and CTQ-SF  *-Roseburia, Bacteroides,* and *Phascolarctobacterium* negatively correlated with HAMD-24 and CTQ-SF  **Mediation analysis**  CTQ-SF had a significant direct effect on HAMD-24, and *Blautia, Bifidobacterium*, and *Roseburia* showed significant indirect effects | | | **Alpha and beta diversity**  ↑ Simpson and Pielou’s evenness in HCs than in the CTQ0 (without childhood maltreatment) group  Significant differences bet Controls and CTQ0 and bet Controls and CTQ1 |
| **68) Zhao et al. (2022)**  Case-control, n=50 (MDD, n=24; Controls, n=26), DOI: 10.1038/s41398-021-01769-x | Metagenomics sequencing analysis-Illumina Novaseq 6000 platform | Diagnosis:  DSM-5  HAMD-17 >17  Symptoms:  HAMD-17, HAMA, 30-item Inventory of Depressive Symptoms-Self Report (IDS-SR30) and the 16-item Quick Inventory of Depressive Symptomatology-Self Report (QIDS-SR16) | **Differential taxa identified by meta stat and LefSe analysis**  *Family level*  ↓ Clostridiaceae (9 species), Bacillaceae (6 species), Ruminococcaceae (4 species), Bacteroidaceae (4 species), and Prevotellaceae (4 species) in MDD  ↑ Bacillaceae (11 species), Bifidobacteriaceae (10 species), Prevotellaceae (7 species), Streptococcaceae (6 species), Lactobacillaceae (5 species), Ruminococcaceae (4 species), and Clostridiaceae (4 species) in MDD  **Random forest analysis to predict biomarkers of MDD**  Best model (AUC was 0.98 (95% CI: 0.961–1)) contained six species:  *Ruminococcus sp CAG: 9 related 41 34,*  *Leptotrichia sp oral taxon 498, Veillonella sp CAG: 933, Sutterella wadsworthensis CAG: 135, Butyrivibrio sp INlla14, Arcobacter lekithochrous*  **Correlations between gut microbiome and clinical characteristic of MDD**  47/94 discriminative species (Ruminococcus being the most important) were highly linked with clinical parameters, such as HAMD-17, HAMA, IDS-SR30, and QIDS-SR16 | | | **Alpha and beta diversity**  Did not analyse |
| **69) Zheng, Yang et al. (2020)**  Case-control, n=599 [MDD, n=165; Bipolar Disorder (BD), n=217;Controls, n=217], DOI: 10.1002/advs.201902862 | Bacteria:  16S rRNA gene sequencing- Illumina MiSeq | Diagnosis:  Structured Clinical Interview for DSM‐IV  Symptoms:  HAMD and Young Manic Rating Scale (YMRS) | **Gut microbial biomarkers for discriminating MDD, BD, Controls (LEfSe and Random Forest analysis)**  *Family level*  Lachnospiraceae (eight OTUs), Bacteroidaceae (seven OTUs), Pseudomonadaceae (three OTUs), and Ruminococcaceae (three OTUs)  *Genus level*  ↑ *Pseudomonas, Ruminococcus_gnavus, Subdoligranulum, Blautia, Pseudomonas, Herminiimonas,*  *Lachnoclostridium, Tyzzerella_4, Pseudomonas,* *Romboutsia* in BD  ↑ *Bacteroides, Erysipelotrichaceae_UCG-003, Fusobacterium* in MDD  ↑ Faecalibacterium, Ruminiclostridium_6, Ruminococcus_gauvreauii, Roseburia, Citrobacter, Coprococcus_2 in Controls | | | **Alpha and beta diversity**  ↓ Ace and Chao in BD vs Controls  No differences bet MDD and BD, or MDD and Controls  BD significantly discriminated from both MDD and Controls; MDD and BD were significantly different from Controls (PLS‐DA) |
| **70) Zheng, Zeng et al. (2016)**  Case-control, n=121 (MDD, n=58; Controls, n=63), DOI: 10.1038/mp.2016.44 | Bacteria:  16S rRNA gene sequencing | Diagnosis:  Structured Psychiatric Interview using DSM-IV-TR criteria Symptoms:  HAMD-17 | **Random forest algorithm carried out to identify the key discriminatory OTUs**  *Phylum level*  ↑ Actinobacteria in MDD  ↓ Bacteroidetes in MDD  Discriminative OTUs were mainly assigned to Firmicutes (45/56, 76.7%), Actinobacteria (5/56, 10.9%) and Bacteroidetes (3/56, 5.3%)  *Order level*  Consistent changes in OTUs assigned to:  ↑ Actinomycetales, Coriobacteriales, Lactobacillales in MDD  ↓ Bacteroidales, Burkholderiales, Selenomonadales in MDD  Classes with changed OTUs in mixed directions: Clostridiales  *Family level*  Consistent changes in OTUs assigned to:  ↑ *Actinomycineae*, *Coriobacteriaceae*, *Erysipelotrichaceae, Eubacteriaceae, Lactobacillaceae*, *Streptococcaceae* in MDD  ↓ *Acidaminococcaceae, Bacteroidaceae, Rikenellaceae, Sutterellaceae,* *Veillonellaceae* in MDD  Families with changed OTUs in mixed directions: *Lachnospiraceae, Ruminococcaceae*  *Genus level*  Consistent changes in OTUs assigned to:  ↑ *Actinomyces, Anaerostipes, Blautia, Clostridium IV*, *Collinsella, Dorea, Eggerthella, Erysipelotrichaceae incertae sedis, Eubacterium, Lactobacillus, Olsenella, Parvimonas, Streptococcus* in MDD  ↓ *Alistipes,* *Bacteroides, Clostridium XIVa*, *Coprococcus, Faecalibacterium, Megamonas, Phascolarctobacterium,* *Sutterella* in MDD  Genera with changed OTUs in mixed directions: *Lachnospiracea incertae sedis, Roseburia* | | | **Alpha and beta diversity**  No significant differences in:  - Richness (observed species)  - Richness/evenness (Shannon, Simpson)  - Phylogenetic diversity  MDD clustered separately from controls on PCoA (unweighted UniFrac, Bray-Curtis) |
| **71) Zheng, Zhu et al. (2021)**  Case-control, n=60 (MDD, n=30; Controls, n=30), DOI: 10.1002/brb3.2036 | Bacteria:  16S rRNA gene sequencing | Diagnosis:  ICD‐10  Symptoms:  HAM-A  HAM-D | **LEfSe**  ↑ *Barnesiella* (genus) in MDD  ↑ Lachnospiraceae (family), *Alloprevotella* (genus), *Clostridium* (species), Succinivibrionaceae (family), Aeromonadales (order) in Controls  **LEfSe on HAMA scores in MDD groups (PG1=with anxiety and PG2=without anxiety)**  ↑ Alcaligenaceae, Burkholderiales, Betaproteobacteria, *Bacteroides massiliensis, Comamonas, Comamonas kerstersii, Mitsuokella*, and *Comamonadaceae* in PG2  ↑ *Facecalibacterium* in PG1 | | | **Alpha and beta diversity**  No significant differences in Chao, ACE, Shannon, Simpson, and Sobe index bet MDD and Controls |
| **72) Zhong et al. (2022)**  Case-control, n=60 (MDD, n=30; Controls, n=30) [MDD subgroups (moderate, n=72; severe, n=58) based on the HAMD] DOI:10.3389/fcimb.2022.907239 | Bacteria:  16S rRNA gene sequencing- Illumina MiSeq PE300 platform/NovaSeq PE250 platform | Diagnosis:  DSM-IV  moderate MDD: HAM-D <25  severe MDD:  HAM-D ≥25 | **Logistic regression adjusting for age, sex, and BMI to identify biomarkers of MDD diagnosis**  ↑ *Collinsella, Eggerthella, Alistipes*, and *Flavonifractor* in MDD  ↑ *Faecalibacterium* in Controls  (AUC = 0.786)  **Correlations between HDRS score and differential genera (Pearson correlation analysis)**  *Moderate MDD*  *Parvimonas, Blautia, Actinomyces,* and *Enterococcus* had significantly negative correlations with HAM-D  *Dorea,* and *Gordonibacter* had significant positive correlations with HAM-D  *Severe MDD*  *Klebsiella, Butyricimonas, Bilophila*, and *Odoribacter* had significantly positive correlations with HAM-D | | | **Alpha and beta diversity**  No significant differences in Shannon and Simpson bet both MDD groups and Controls  Significant differences on β-diversity bet Controls and severe MDD as well as Controls and moderate MDD |
| **73) Zhu et al. (2022)**  Case-control, n=60 (Anxiety and depressive symptoms, n=30; Controls, n=30), DOI: 10.3389/fpsyt.2021.757139 | Bacteria:  16S rRNA gene sequencing | Symptoms:  GAD-7 (anxiety)  PHQ-9 (depression) | *Phylum level*  ↑ Bacteroidetes and Proteobacteria in patients with anxiety and depression  ↓ Firmicutes and Actinobacteria decreased in patients with anxiety and depression, although there was no significant difference.  **LEfSe**  ↑ *Pediococcus, Erysipelatoclostridium, Granulicatella, Kluyvera, Shuttleworthia, Vagococcus,* and *Faecalicatena* in screeners with anxiety/depression  ↓ *Gemmiger, Veillonella, Ruminococcus, Anaerovorax*, and *Barnesiella* in screeners with anxiety/depression | | | **Alpha and beta diversity**  No significant differences in evenness, observed OTUs, Shannon and Chao1  Significant clustering bet the anxiety/depression and control group (Jaccard but not Bray-Curtis, weighted& unweighted unifrac) |

↑ = higher relative to comparison group; ↓ = lower relative to comparison group; ↔ = inconsistent reporting or unable to decipher direction of relationship

BAI = Beck Anxiety Inventory; BDI = Beck Depression Inventory; DASS = Depression Anxiety Stress Scale; FGFP = the Flemish gut flora project; GAD = Generalised Anxiety Disorder; HAM-D = Hamilton Depression Rating Scale; HAM-A = Hamilton Anxiety Rating Scale; IBS = irritable bowel syndrome; IBS-D = IBS-diarrhoea predominant; LEfSe = Linear discriminant analysis Effect Size; MADRS = Montgomery-Asberg Depression Rating Scale; MDD = major depressive disorder; OTUs = observed taxonomic units; PCoA = principal coordinates analysis; PERMANOVA = permutational analysis of variance; PLS-DA = principal least squares discriminant analysis;  qPCR = quantitative polymerase chain reaction; SAD = Social Anxiety Disorder; SAS = self-rating anxiety scale; SDS = self-rating depression scale


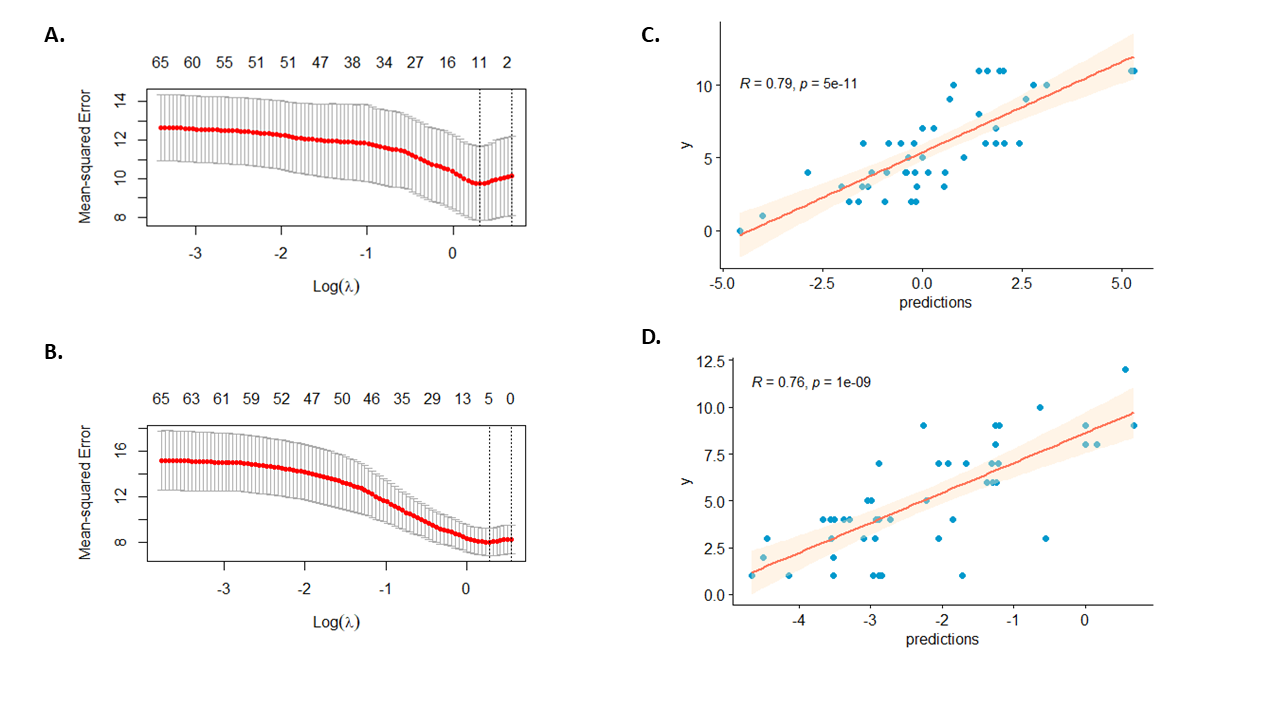


**Figure S2.** Cross-validation Mean Square Error curve for the: A. anxiety and B. depressive symptoms model and predictions plots for the: C. anxiety and D. depressive symptoms model.


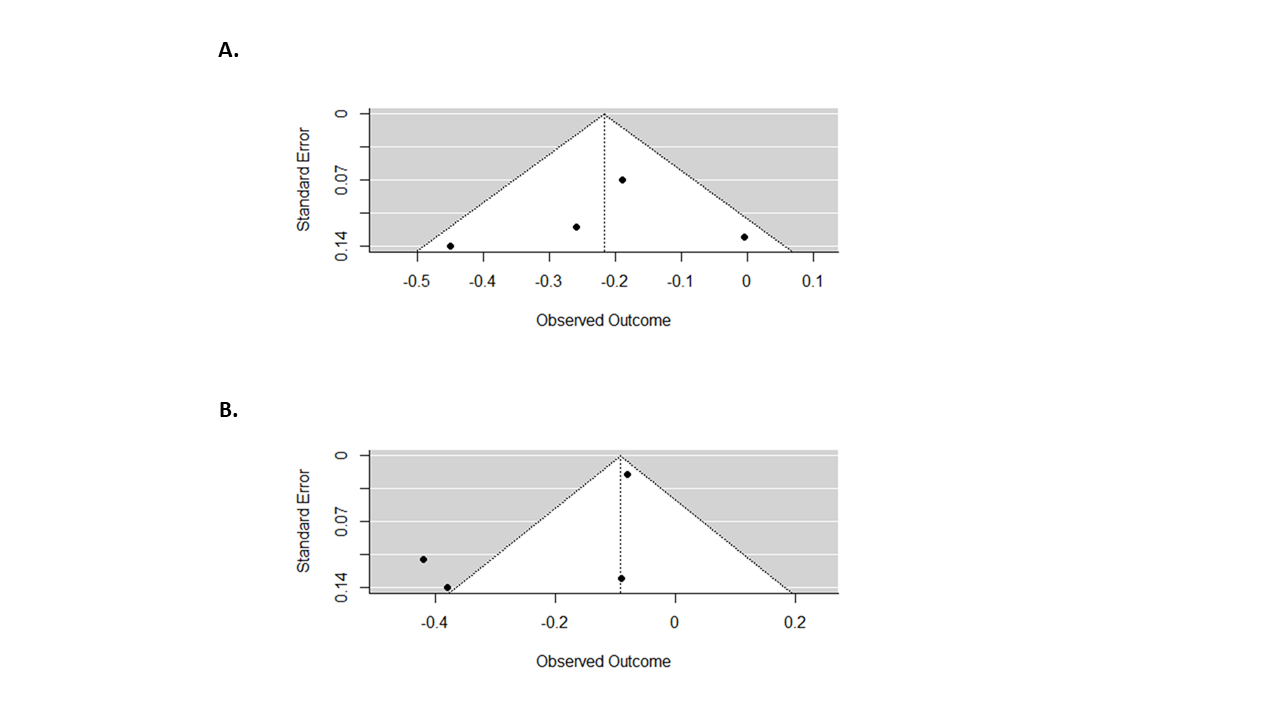


**Figure S3.** Funnel plots for publication bias for the meta-analysis of: A. Bifidobacterium with anxiety symptoms and B. Lachnospiraceae NK4A136 group with depressive symptoms.

**Table S2.** Linear regressions with anxiety and depressive symptom levels adjusted for pain medication use (NSAIDs and Opioids)

| **Anxiety symptoms** | **Discovery (OA1) (n = 46)** | | | **Replication (OA2) (n = 67)** | | |
| --- | --- | --- | --- | --- | --- | --- |
|  | **Beta** | **SE** | **p-value** | **Beta** | **SE** | **p-value** |
| *Bifidobacterium* | -0.47 | 0.14 | 0.001 | -0.27 | 0.12 | 0.031 |
| *Clostridium sensu stricto 1* | -0.39 | 0.14 | 0.011 | -0.27 | 0.12 | 0.034 |
| *Coprococcus* | -0.35 | 0.15 | 0.024 | -0.26 | 0.12 | 0.037 |
| *Bacteroides* | -0.41 | 0.15 | 0.010 | -0.12 | 0.13 | 0.339 |
| *Anaerostipes* | 0.38 | 0.15 | 0.015 | 0.14 | 0.13 | 0.282 |
| *Blautia* | 0.36 | 0.15 | 0.025 | 0.13 | 0.13 | 0.307 |
| Depressive symptoms |  |  |  |  |  |  |
| *Butyricicoccus* | 0.40 | 0.14 | 0.008 | 0.10 | 0.13 | 0.418 |
| *Lachnospiraceae NK4A136 group* | -0.33 | 0.14 | 0.024 | -0.43 | 0.12 | 0.0005 |
| *Roseburia* | -0.33 | 0.14 | 0.023 | -0.20 | 0.12 | 0.110 |

**Table S3.** Mean relative abundances (%) of signature taxa that were significant after adjusting for covariates in the Discovery cohort

| **Taxa** | **Discovery (OA1) (n = 46)** | **Replication (OA2) (n = 67)** | **Replication (CON) (n = 58)** |
| --- | --- | --- | --- |
|  | **% Mean relative abundance ± SD** | **% Mean relative abundance ± SD** | **% Mean relative abundance ± SD** |
| *Bifidobacterium* | 0.61 ± 2.67 | 0.52 ± 0.97 | 0.17 ± 0.67 |
| *Clostridium sensu stricto 1* | 0.02 ± 0.07 | 0.40 ± 0.65 | 0.10 ± 0.29 |
| *Coprococcus* | 0.63 ± 1.72 | 0.19 ± 0.51 | 0.02 ± 0.02 |
| *Anaerostipes* | 0.41 ± 0.95 | 1.30 ± 1.50 | 0.03 ± 0.06 |
| *Lachnospiraceae NK4A136 group* | 0.13 ± 0.28 | 0.07 ± 0.37 | 0.48 ± 0.42 |
